# Design of high avidity and low affinity antibodies for in situ control of antibody drug conjugate targeting

**DOI:** 10.1038/s41598-022-11648-0

**Published:** 2022-05-10

**Authors:** Reginald Evans, Greg M. Thurber

**Affiliations:** 1 Department of Chemical Engineering, University of Michigan, 2800 Plymouth Rd., Ann Arbor, MI 48109 USA; 2 Department of Biomedical Engineering, University of Michigan, Ann Arbor, MI 48109 USA; 3 Rogel Cancer Center, University of Michigan Medicine, Ann Arbor, MI 48109 USA

**Keywords:** Drug development, Computational models, Drug delivery

## Abstract

Antibody-Drug Conjugates (ADCs) have rapidly expanded in the clinic, with 7 new approvals in 3 years. For solid tumors, high doses of ADCs improve tissue penetration and efficacy. These doses are enabled by lower drug-to-antibody ratios and/or co-administration of unconjugated antibody carrier doses to avoid payload toxicity. While effective for highly expressed targets, these strategies may not maintain efficacy with lower target expression. To address this issue, a carrier dose that adjusts binding in situ according to cellular expression was designed using computational modeling. Previous studies demonstrated that coadministration of unconjugated antibody with the corresponding ADC at an 8:1 ratio improves ADCs efficacy in high HER2 expressing tumors. By designing a High Avidity, Low Affinity (HALA) carrier antibody, ADC binding is partially blocked in high expression cells, improving tissue penetration. In contrast, the HALA antibody cannot compete with the ADC in low expressing cells, allowing ADC binding to the majority of receptors. Thus, the amount of competition from the carrier dose automatically adjusts to expression levels, allowing tailored competition between different patients/metastases. The computational model highlights two dimensionless numbers, the Thiele modulus and a newly defined competition number, to design an optimal HALA antibody carrier dose for any target.

## Introduction

Antibody Drug Conjugates (ADCs) are a rapidly growing class of therapeutics for the treatment of cancer with 7 new approvals in the past 3 years. There are currently 11 approved ADCs and hundreds more in clinical or preclinical development^1^. ADCs are composed of an antibody that binds to cancer cells and a cytotoxic payload connected via a linker^2^. These therapeutics are advantageous because they combine the high specificity of antibodies with the cell killing potential of chemotherapy. However, these agents also combine the challenges of delivering macromolecules to tumors with the toxicity of small molecules, resulting in many failures over the past two decades^3,4^. This leads to challenges in ADC development because of variability in the tumor microenvironment and a generally narrow therapeutic index^5^.

There are many strategies to optimize antibodies and ADC chemical properties to expand the therapeutic window and improve efficacy. Antibodies and ADCs distribute heterogeneously within the tumor microenvironment both as a function of target expression and rapid binding within the tissue (binding site barrier effect)^6–8^. While increasing the dose is a well-known technique for more homogenous distribution of antibodies, ADC doses are limited by toxicity due to their payload^9,10^. One method for expanding the therapeutic window is to decrease the potency of ADCs for high expression targets^11^. This enables higher dosing that can increase the tissue penetration of the ADC in the tumor, reaching and killing more cells, while also lowering the target-mediated uptake of ADCs in healthy tissue, reducing toxicity^11^. Another strategy is using site specific conjugation, where the payload is conjugated to a specific site of the antibody using a chemical or enzymatic methods^12–14^. Site specific conjugation has allowed for more homogenous ADCs which can show better plasma stability, less variability in dose response and less toxicity compared to conventional ADCs for in vivo studies^9,15–18^. A third strategy is modifying the binding affinity of the ADC or utilizing lower molecular weight compounds with faster diffusion in tissue^19–21^. The specificity of the ADC to its target cells can also be modified^22–24^, such as bispecific agents^25^ to control tumor distribution.

One method to reduce potency and increase tissue penetration is coadministration of the unconjugated antibody with the corresponding ADC. For Kadcyla, a ratio of up to 8:1 trastuzumab to Kadcyla dose improves ADC efficacy in xenograft tumors^26–28^. Because the unconjugated antibodies are administered at a higher dose, they compete with the ADC and partially block the cell receptors, allowing the ADCs to penetrate deeper into the tumor to improve efficacy. However, many carrier dose studies focused on high expression targets (10^6^ receptors/cell). For lower expression level targets, the benefit of a carrier dose is diminished, and at a low enough expression, efficacy is reduced if not enough payload can enter the cell for cell death (e.g. (10^4^–10^5^ receptors/cell))^29^.

**Figure 1:**
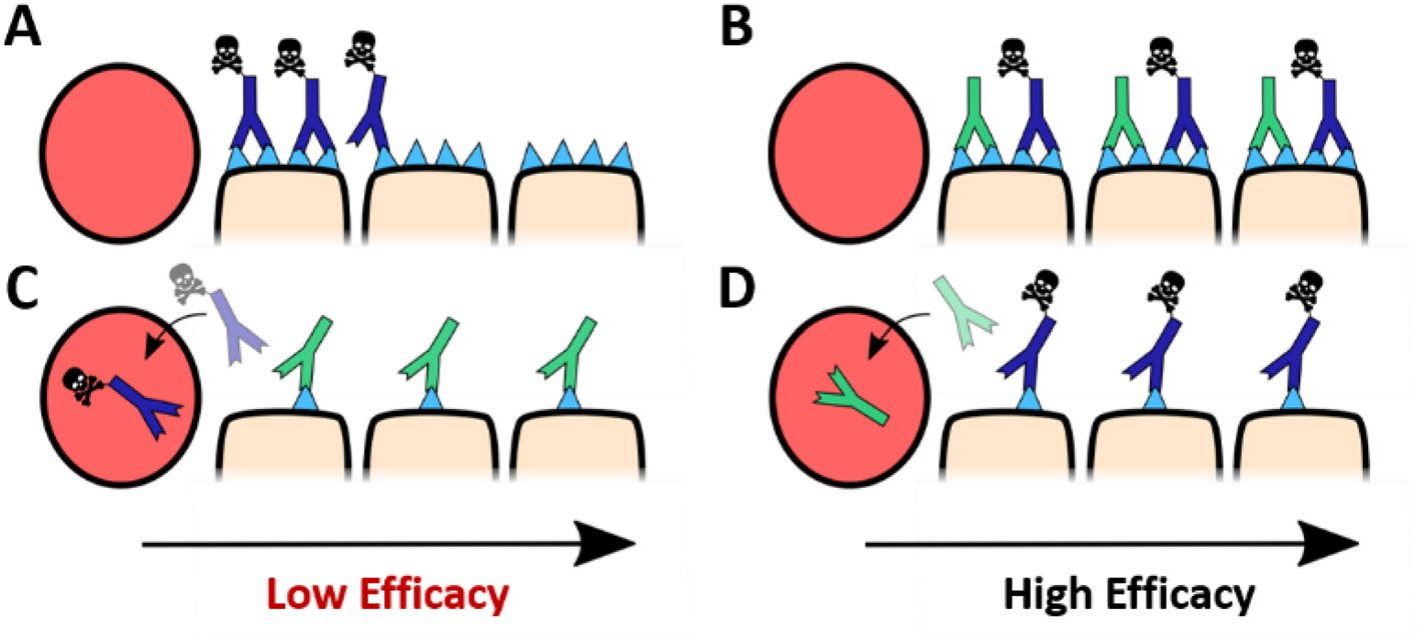
Improving ADC distribution through coadministration of HALA antibody carrier doses. Administration of an ADC (single agent) produces heterogeneous perivascular distribution due to rapid binding relative to transport in the tissue in high expression tumors (e.g. at 3.6 mg/kg for Kadcyla, (**A**). Tumor penetration is improved when the ADC is co-administered with a saturating dose of unconjugated antibody (e.g. 8:1 ratio of antibody to ADC, (**B**). The two antibodies compete for binding sites increasing ADC penetration. However, in low expression systems, unconjugated antibodies can outcompete the ADC (**C**), lowering efficacy. Coadministration of a HALA antibody allows competition in high expression systems (**B**) while enabling the ADC to outcompete the HALA antibody in low expression systems (**D**).

In this work, a novel carrier antibody engineered for High Avidity and Low Affinity (HALA) is simulated to determine if it can overcome this limitation by in situ adjustment of competitive blocking in the tumor microenvironment. HALA antibodies have a weaker monovalent binding affinity than ADCs; therefore, the HALA antibodies are hypothesized to compete with the ADCs in tumors with high expression where avidity is strong, pushing the ADCs farther into the tumor to improve efficacy. However, in low expression tumors, where the avidity effect is weaker, the ADC will outcompete the HALA antibody, resulting in high ADC binding and efficacy. Essentially, the HALA antibody is capable of automatically adjusting its competition within different metastases in the same patient, or even cells within the same tumor, to maximize efficacy. A diagram of this model can be seen in Fig. 1.

To guide the development of HALA antibodies, the HALA competition with an ADC was simulated using validated mechanistic models of bivalent binding in monolayer cells, in tumor spheroids, and in vivo using a Krogh Cylinder model. The results provide insight into the dependency of ADC distribution on co-administered HALA antibody binding affinity, receptor expression, and internalization rate. The simulation results can be used to optimize the design of the co-administered HALA antibody affinity with ADCs to enable maximum efficacy independent of expression heterogeneity.

## Material and methods

Three previously validated computational models of increasing complexity^30–32^ were used to describe the cellular, tissue, and in vivo impact of HALA antibody properties. The first model is a bivalent competition kinetic model used to measure HALA antibody competition versus the ADC on a cell monolayer. The next model incorporated the competition kinetics alongside target internalization and antibody diffusion in the simulation. This model varied HALA antibody binding affinity and concentration in tumor spheroids to determine maximum ADC efficacy as a function of binding affinity. Finally, the competition kinetic model was integrated into a Krogh Cylinder model. The Krogh Cylinder model is a multiscale model that incorporates Pharmacokinetic/Pharmacodynamic (PK/PD) phenomena, such as transport limited by permeability, plasma clearance, payload release, and bystander killing, where the payload is free to diffuse into adjacent cells. This model was used to simulate antibody competition and payload distribution as a function of tumor expression and HALA antibody properties.

### Monolayer competition model

**Figure 2:**
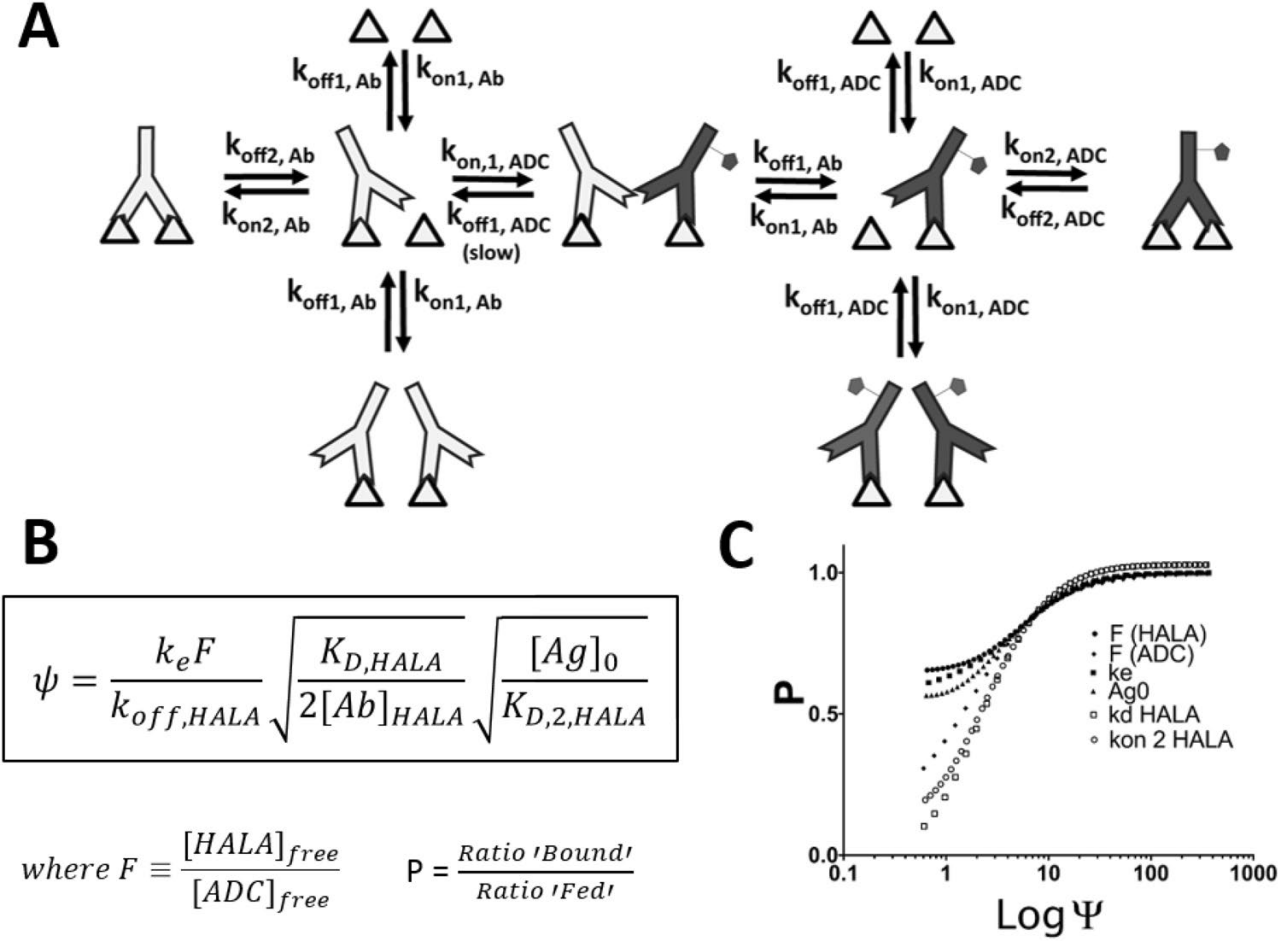
Competition kinetics between ADCs and HALA antibodies. The reaction network of ADC (gray) and HALA antibodies (white) are shown in (**A**). Two antigens (triangles) are shown to emphasize the bivalent interactions. A dimensionless ‘competition’ number, Ψ, is defined based on assuming irreversible binding of the high affinity ADC (**B**). The ratio of bound HALA antibody and ADC relative to the ratio of HALA antibody and ADC in the bulk media (P) versus Ψ shows that at Ψ values of 10 or more, the HALA antibody successfully competes for binding with the ADC. The dimensionless number captures the competition rate independent of which parameters are varied (**C**). For lower values of Ψ, the HALA antibody cannot compete with the ADC, and the bound fraction of ADC is higher than the fraction in solution (low P). Because Ψ is a function of expression, HALA antibodies can be designed to have a large Ψ value for high expressing cells and small Ψ for low expressing cells.

A computational model for competition between two bivalent antibodies was developed by adapting a kinetic model^33,34^ to simulate HALA antibody competition with ADCs on a monolayer. Briefly, the HALA antibody and ADC compete for open binding sites. The monovalently bound HALA antibody or ADC can dissociate or bind bivalently. At the same time, the free binding sites are in competition with new HALA antibodies or ADCs binding to the target. A summary of this phenomenon is depicted in Fig. 2A.

The differential equations were defined from the reaction network, and initial competition parameters were calculated or determined from the literature:

### Reaction network

1 $$ \frac{{d\left[ {Ab_{ADC} } \right]}}{dt} = - 2k_{{on\left( {1ADC} \right)}} \left[ {Ag} \right]\left[ {Ab_{ADC} } \right] + k_{{off\left( {1ADC} \right)}} \left[ {M_{ADC} } \right] $$

2 $$ \frac{{d\left[ {Ab_{HALA} } \right]}}{dt} = - 2k_{{on\left( {1HALA} \right)}} \left[ {Ag} \right]\left[ {Ab_{HALA} } \right] + k_{{off\left( {1HALA} \right)}} \left[ {M_{HALA} } \right] $$

Monovalent Binding event

3 $$ \begin{aligned} \frac{{d\left[ {M_{ADC} } \right]}}{dt} = & \,2k_{{on\left( {1ADC} \right)}} \left[ {Ag} \right]\left[ {Ab_{ADC} } \right] - k_{{off\left( {1ADC} \right)}} \left[ {M_{ADC} } \right] \\& - k_{{on\left( {2ADC} \right)}} \left[ {M_{ADC} } \right]\left[ {Ag} \right] + 2k_{{off\left( {2ADC} \right)}} \left[ {B_{ADC} } \right] - k_{e} \left[ {M_{ADC} } \right] \\ \end{aligned} $$

4 $$ \begin{aligned} \frac{{d\left[ {M_{HALA} } \right]}}{dt} = & \,2k_{{on\left( {1HALA} \right)}} \left[ {Ag} \right]\left[ {Ab_{HALA} } \right] - k_{{off\left( {1HALA} \right)}} \left[ {M_{HALA} } \right] - k_{{on\left( {2HALA} \right)}} \left[ {M_{HALA} } \right]\left[ {Ag} \right] \\ & + 2k_{{off\left( {2HALA} \right)}} \left[ {B_{HALA} } \right] - k_{e} \left[ {M_{HALA} } \right] \\ \end{aligned} $$

Bivalent Binding event

5 $$ \frac{{d\left[ {B_{ADC} } \right]}}{dt} = k_{{on\left( {2ADC} \right)}} \left[ {M_{ADC} } \right]\left[ {Ag} \right] - 2k_{{off\left( {2ADC} \right)}} \left[ {B_{ADC} } \right] - k_{e} \left[ {B_{ADC} } \right] $$

6 $$ \frac{{d\left[ {B_{HALA} } \right]}}{dt} = k_{{on\left( {2HALA} \right)}} \left[ {M_{HALA} } \right]\left[ {Ag} \right] - 2k_{{off\left( {2HALA} \right)}} \left[ {B_{HALA} } \right] - k_{e} \left[ {B_{ADC} } \right] $$

Antigen Balance

7 $$ \begin{aligned} \frac{{d\left[ {Ag} \right]}}{dt} = & - 2k_{{on\left( {1ADC} \right)}} \left[ {Ag} \right]\left[ {Ab_{ADC} } \right] + k_{{off\left( {1ADC} \right)}} \left[ {M_{ADC} } \right] - 2k_{{on\left( {1HALA} \right)}} \left[ {Ag} \right]\left[ {Ab_{HALA} } \right] \\ & + k_{{off\left( {1HALA} \right)}} \left[ {M_{HALA} } \right] - k_{{on\left( {2ADC} \right)}} \left[ {M_{ADC} } \right]\left[ {Ag} \right] \\ & - k_{{on\left( {2HALA} \right)}} \left[ {M_{HALA} } \right]\left[ {Ag} \right] + 2k_{{off\left( {2ADC} \right)}} \left[ {B_{ADC} } \right] \\ & + 2k_{{off\left( {2HALA} \right)}} \left[ {B_{HALA} } \right] + R_{s} - k_{e} \left[ {Ag} \right] \\ \end{aligned} $$

Receptor Synthesis Rate for Steady State Surface Concentration

$$ R_{s} = k_{e} \left[ {Ag} \right]_{0} $$

where [Ab_ADC/HALA_] is the ADC or HALA concentration respectively, k_on(1ADC/HALA)_ is the on rate of the first binding arm of the ADC or HALA antibody respectively, k_off(1ADC/HALA)_ is the off rate of the HALA Antibody or ADC, $\left[ {Ag} \right]$ is the concentration of free cell receptors, [M_ADC/HALA_]is the concentration of the Monovalently bound ADC or HALA complex, [B_ADC/HALA_] is the concentration of the Bivalently bound ADC or HALA complex, k_on(2ADC/HALA)_ is the on rate of the second binding arm of the ADC or HALA and $k_{e}$ is the internalization rate.

Full descriptions of the constants and variables used in the model is defined in Supplementary TABLE S1 and S2.

### Spheroid competition model

The bivalent competition model was adapted to spherical geometry with diffusion using a previous validated model to simulate ADC distribution in spheroids^2^. Differential equations, parameters and variables are shown in the supplementary materials.

### Krogh cylinder model

The spheroid model does not capture vascular permeability^33^, plasma clearance, and payload distribution following release. Therefore, the competition model was adapted to a previously validated Krogh Cylinder geometry to simulate ADC and Payload distribution in vascularized tumors^5,11,25,28,34,35^. The Krogh Cylinder model assumes radial but not axial distribution because concentration varies little along the capillary length^36^. Elevated interstitial pressure results in limited convection and primarily diffusive transport in the tissue^37^.

In this model, the antibody and ADC compete for available binding sites as above. Upon binding to a cell receptor, the ADC (or HALA antibody) is internalized into the cell, and the Antibody is degraded, allowing the payload to kill cells. Two types of payloads are included: hydrophilic and lipophilic payloads. Hydrophilic payloads are trapped within the targeted cell, while lipophilic payloads can diffuse outside the cell and into surrounding cells (the bystander effect).

## Results

The model was validated by simulating the amount of bound IgG for affinity variants to cells with different levels of cellular expression (Supplementary Fig. S1^38^). After validation, an expression “Ψ” was derived to describe effective competition of the HALA antibody. It is essentially the ratio between cellular receptor internalization relative to binding competition, Fig. 2B.

### HALA competition dimensionless number

A simplified description of competition kinetics between ADC and HALA antibodies was developed where binding of the ADC was assumed to be irreversible (high affinity). A dimensionless number, Ψ, was derived to capture the ability of the HALA antibody to compete with the higher affinity ADC (Supplementary Data).

Within minutes of adding the antibodies in silico, both the ADC and HALA antibody significantly bind the target. Since k_on_ rates are similar for many antibodies (and k_off_ typically distinguishes high versus low affinity antibodies), the amounts of bound ADC and HALA antibody are proportional to their concentrations in solution (Supplementary Fig. S2). Over the course of hours, the higher affinity ADC starts to outcompete the HALA antibody, increasing the amount of ADC bound. However, once the receptor is internalized, the ADC can no longer compete for binding. The rate of competition of ADC relative to the rate of internalization is expressed as Ψ, which is dependent on the receptor expression level. At low Ψ values, there is poor HALA antibody binding, and the ADC outcompetes the HALA for binding sites. At high Ψ, the HALA antibody is able to compete with the ADC until the antigen is internalized.

To validate the dimensionless number, numerical simulations were performed, and Ψ was graphed versus P, the amount of HALA antibody bound relative to the initial concentration. When P = 1, the amount of HALA binding is proportional to the initial dose, so at an 8:1 HALA antibody to ADC ratio, the HALA antibody binds 8 times more receptors than the ADC. If P is less than 1, the ADC is outcompeting the HALA antibody relative to the initial concentration of each agent, and if P is greater than 1, HALA binding out-competes the ADC (not shown, since this implies a higher affinity HALA antibody). Each parameter of Ψ was varied to validate the relationship between Ψ and P. Regardless of the individual parameter changed, at high values of Ψ, the *P* value converges to 1. At low values of Ψ, the *P* value varies depending on the conditions (Supplementary Fig. S3). Results are displayed in Fig. 2C.

### Modeling spheroids

**Figure 3:**
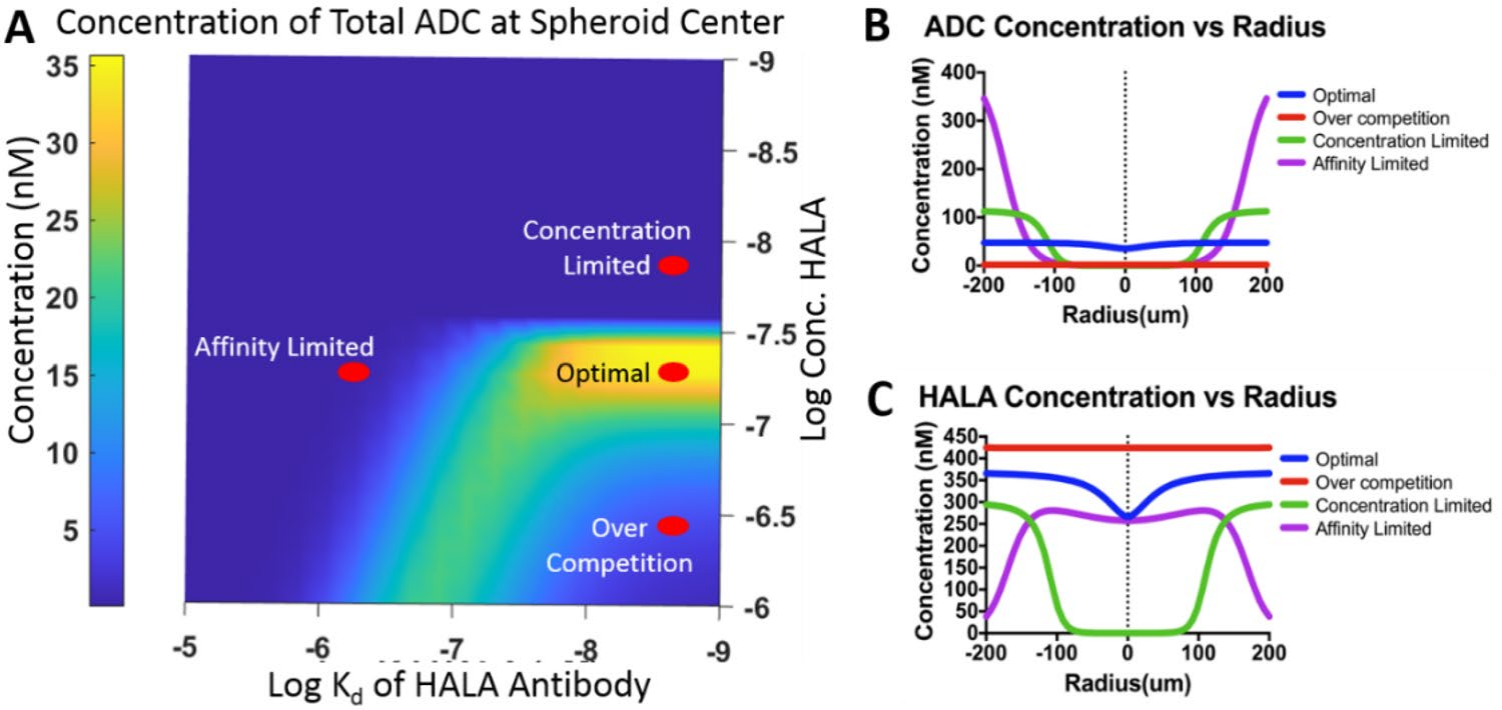
Simulating ADC distribution in spheroids. (**A**) Spheroid model simulating the concentration of ADC binding at the center of the spheroid. Both the affinity of the HALA antibody and the concentration of HALA antibody were varied. As the affinity of the HALA antibody is reduced, higher concentrations of HALA antibody are required to maintain competition and drive ADC to the spheroid center. Concentration of the ADC binding (**B**) or HALA antibody binding (**C**) over varying distances within the spheroid.

Previous work demonstrated that a co-administered dose of trastuzumab with T-DM1 at an 8:1 ratio allowed TDM-1 to penetrate farther into a tumor for optimal in vivo efficacy^26^. In this work, the affinity of the competitive antibody was varied to determine the impact on tissue penetration and cellular uptake under different levels of expression. Note that the concentration plotted is for cells at the spheroid center, which is typically lower than peripheral cells. The simulations show that most ADC reaches the center of the spheroid at a binding affinity between 1 and 20 nM and a concentration of initial HALA antibody of 40 nM to 80 nM, with an optimum penetration at 5 nM K_d_ and HALA antibody concentration of 40 nM (Fig. 3A). This base case condition, using the affinity and dose of trastuzumab shown to work well in vivo, is labeled “optimal”. Maintaining the same binding affinity as the “optimal” conditions, but increasing or decreasing the HALA antibody concentration, resulted in decreased ADC penetration, which is labeled “over competition” (too large of a carrier dose) or “concentration limited” (too little carrier dose). Maintaining an optimal concentration but decreasing the binding affinity resulted in low penetration, labeled “affinity limited”. A more generalizable plot of ADC concentration at the spheroid center is shown in Supplementary Fig. S4, where the value is plotted versus Ψ and the Thiele modulus, φ^2^. Dimensional values (i.e. concentration and K_d_) are often more intuitive and are therefore used throughout the main figures.

Figure 3B,C show the penetration of the ADC and HALA antibody inside the spheroid as a function of distance/radius. The affinity-limited point shows high ADC concentration at the edge of the spheroid (more than needed for cell death), but the concentration rapidly decreases closer to the center of the spheroid resulting in limited efficacy. Under these conditions, the very low affinity HALA antibody is outcompeted at the edges of the spheroid but reaches the center. In contrast, the model demonstrates homogeneous ADC distribution at the optimal conditions. If the carrier dose is too high, the over-competition point shows a low ADC concentration due to the large dose of HALA antibody. The HALA antibody concentration is large and homogeneous throughout the spheroid. The concentration-limited point shows slightly improved ADC penetration at the edge of the spheroid; however, penetration declines beyond 100 microns. The HALA antibody also lacks tissue penetration.

### Modeling Krogh cylinder

**Figure 4:**
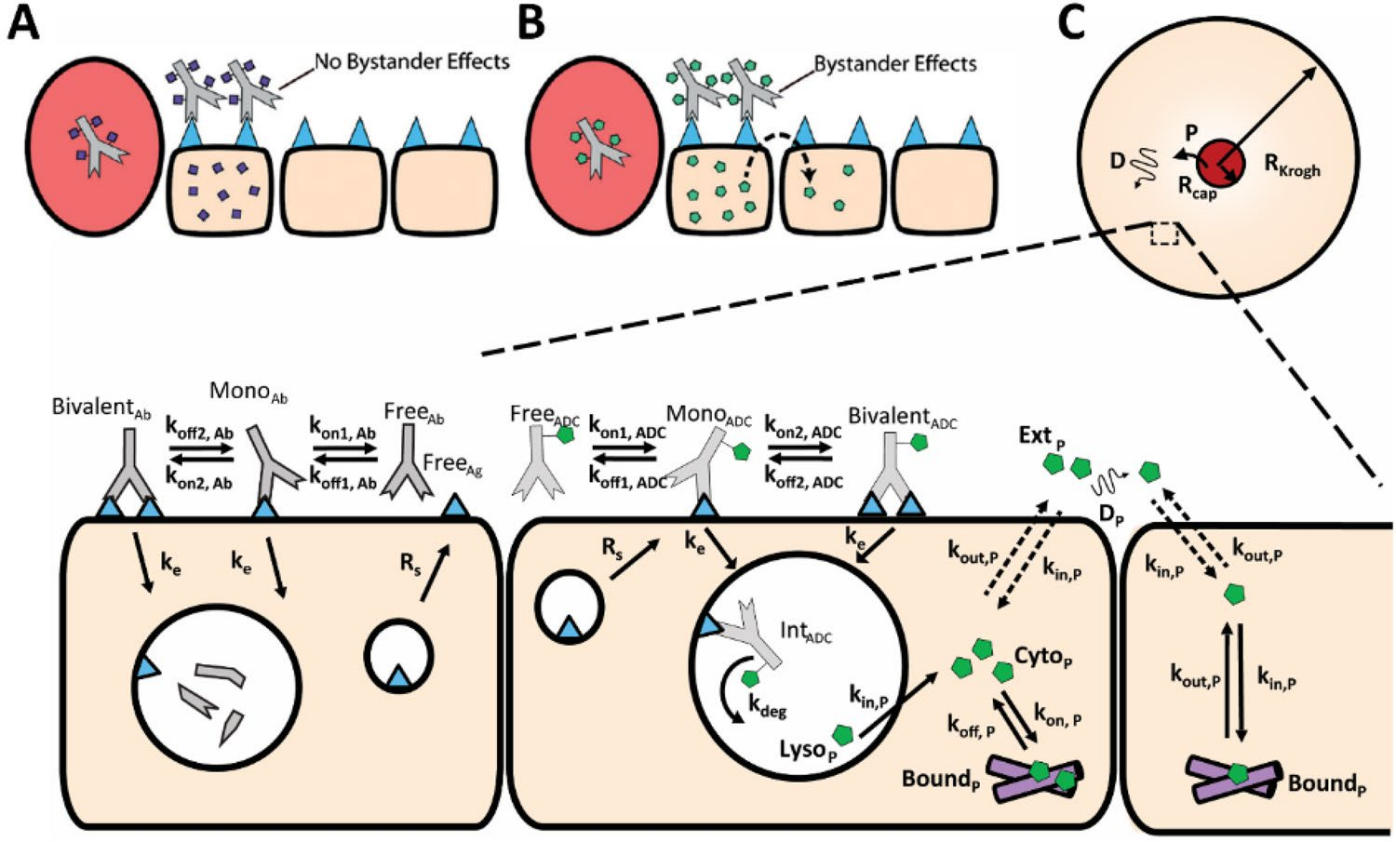
Simulating distribution of ADC payloads in vivo. (**A**) Schematic highlighting non-bystander payloads and cell killing with lysine-SMCC-DM1 (maytansinoid with a non-cleavable linker). (**B**) Schematic highlighting bystander penetration into surrounding cells using MMAE (monomethyl auristatin E). (**C**) Diagram of competition reactions between ADC and HALA antibody and payload distribution in the Krogh Cylinder Model.

After examining the impact of HALA antibody properties on the distribution of ADCs in spheroids, ADC distribution was simulated in vivo using a Krogh Cylinder model. This model included vascular permeability, plasma clearance, and bystander payload distribution (Fig. 4).

The starting simulations used the Krogh Cylinder model and varied the binding affinity and concentration of the HALA antibody similar to the spheroid model to capture the distribution of the ADC and cytotoxic payload. The model first captures an ADC with the cytotoxic payload released by Kadcyla, lysine-SMCC-DM1 (referred to as DM-1), which is a non-bystander payload. Next, the model simulated an ADC with MMAE, a bystander payload, with both results shown 48 h after coadministration.

**Figure 5:**
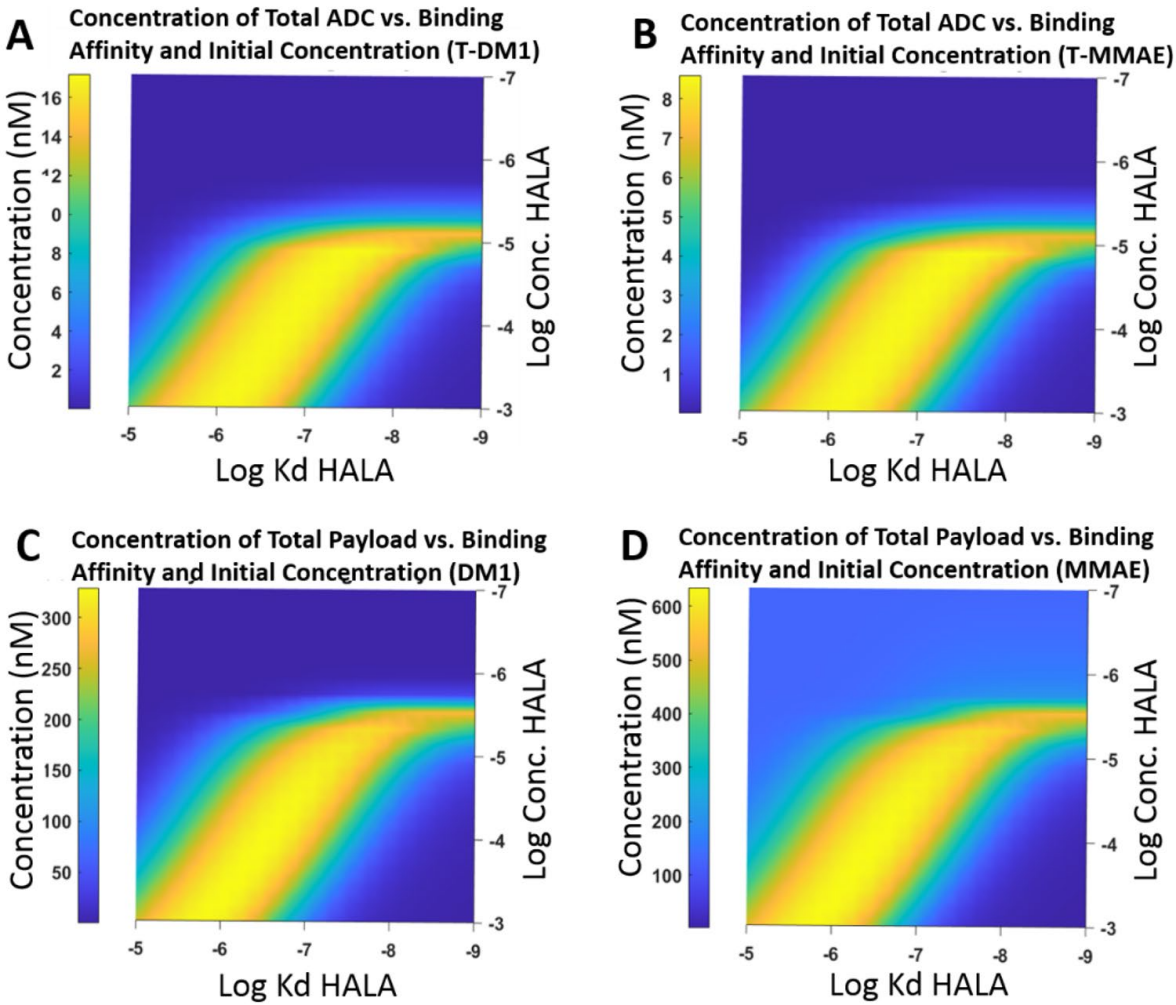
Distribution of ADC and Payload of co-administered ADC with HALA antibody showing changing concentration and binding affinity in Krogh Cylinder simulations after 48 h. (**A**) Distribution of ADC at a dose of 3.6 mg/kg with DM1 payload (non-bystander) (**B**) Distribution of ADC at a dose of 1.8 mg/kg with bystander (MMAE) payload. (**C**) Payload distribution of ADC with non-bystander payload. (**D**) Payload distribution of ADC with bystander payload. The lighter blue region above the yellow curve in D has a higher concentration due to MMAE bystander payload distribution. Concentrations are shown at the Krogh cylinder radius, R_Krogh_, far from tumor blood vessels.

As seen in Fig. 5, the ADC distribution is impacted by both binding affinity and concentration of the HALA antibody. As the initial concentration of the HALA antibody in the plasma increases, the binding affinity must decrease to compensate and maintain good penetration into the tumor. Differences between ADC and Payload distribution after 48 h can be attributed to differences in internalization (Supplementary Figs. S5 and S6). Supplementary Fig. S7 demonstrates a more generalizable plot of ADC and Payload concentration as function of Ψ and φ^2^.

### Designing optimal HALA antibodies

The goal of using HALA antibodies as a carrier dose is to enable efficient penetration in high expression tumors while avoiding competition in cells, tumors, and patients with lower receptor expression. Therefore, from a design perspective, the optimal affinity should be selected based on uptake across a range of receptor expression levels. To accomplish this, the payload distribution was simulated as a function of affinity and receptor expression to select an optimal affinity. The dose was fixed at an 8:1 HALA to ADC ratio because previous experiments indicated this was an optimal dosing ratio in vivo when targeting HER2 using Kadcyla in a high expression tumor (based on tolerability of the ADC). The optimal ratio for other systems will depend on the maximum tolerated dose of the ADC and required antibody dose to saturate the tumor. The binding affinity of the HALA antibody was varied from 1 nM to 10 μM, and the expression ranged between 10,000 rec/cell to 1,000,000 rec/cell.

**Figure 6:**
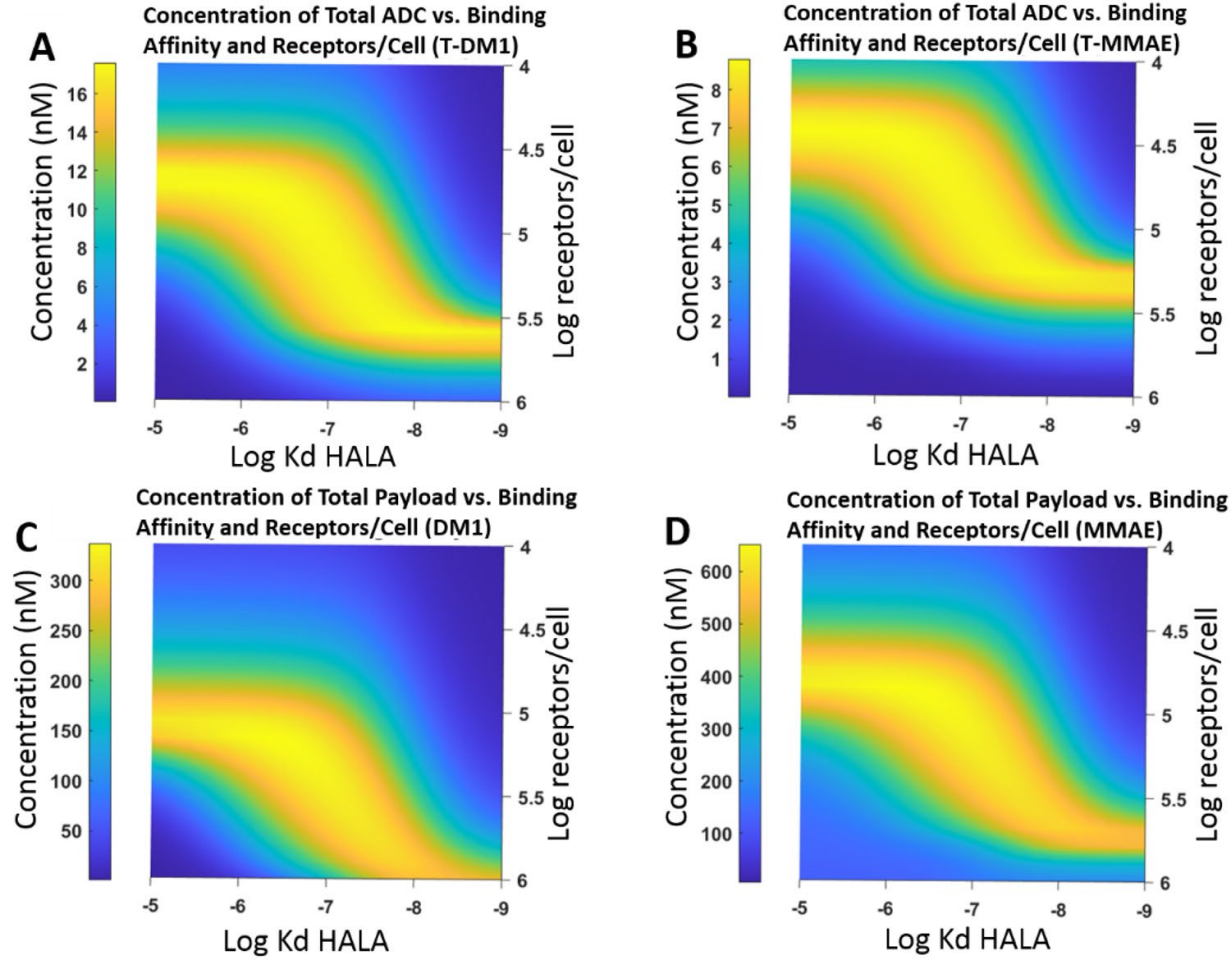
Design of optimal HALA with changing antibody dose and affinity after 48 h. Distribution of ADC and Payload of coadministered ADC HALA antibody with changing concentration and tumor expression in Krogh Cylinder simulations after 48 h. (**A**) Distribution of ADC at a dose of 3.6 mg/kg with DM1 payload (non-bystander) (**B**) Distribution of ADC at a dose of 1.8 mg/kg with bystander (MMAE) payload. (**C**) Payload distribution of ADC with non-bystander payload. (**D**) Payload distribution of ADC with bystander payload. Difference between ADC and Payload distribution is due to the ADC mostly being cleared at the end of the 48 h; however, payload is internalized and retained. Concentrations are shown at the Krogh cylinder radius, R_Krogh_, far from tumor blood vessels.

Figure 6 shows the distribution of the ADC reaching cells far from the tumor vasculature (at the edge of the Krogh cylinder). Stronger binding HALA antibodies increased penetration into high expression tumors, while more weakly bound HALA antibodies were optimal for low expression levels. Around 20 nM, however, the HALA antibody was able to increase uptake in high expression tumors while maintaining payload uptake in moderate expressing tumors. Figure 6B shows the ADC with MMAE as the payload. It demonstrates similar behavior to the DM1 case; however, the 8:1 ratio is not sufficient to enable full penetration at a 1.8 mg/kg ADC dose, so maximum ADC binding occurs at fewer receptors/cell. Based on these graphs, an optimal HALA antibody affinity can be selected. By appropriately choosing the HALA antibody properties, competition on high expressing cells is efficient, improving tissue penetration several-fold. However, competition on low expressing cells is poor, enabling a majority of receptor to be bound by ADC despite an eightfold higher concentration of HALA antibody in solution. Supplementary Figure **S8** demonstrates a more generalizable plot of ADC and Payload concentration as function of Ψ and φ^2^.

## Discussion

Poor tissue penetration of ADCs is a major limitation to their efficacy and has hampered wider clinical success of ADCs^11,26,34,36,37^. Higher antibody doses, whether via lower DAR^37,39,40^, more tolerable payloads^41^, or adding carrier doses^11,26,27,42^ can improve the efficacy while lowering target-mediated uptake in healthy tissue to improve the therapeutic window. However, the lower (effective) DAR can also reduce efficacy in lower expression models^11,43,44^. To overcome this limitation, the distribution of a High Avidity, Low Affinity (HALA) carrier antibody was simulated to determine its impact on distribution. The simulations indicate this type of antibody could improve tissue penetration in high expression models while not significantly competing with the ADC in tumors or cells that have lower expression (Fig. 1). This approach could pair synergistically with other strategies such as higher potency, site-specific ADCs to target these lower expression cells.

A simple conceptual picture shows that a HALA antibody binds bivalently (with high avidity) when target expression is high, enabling it to compete with the high affinity ADC. At low expression levels, the HALA antibody binds monovalently (low avidity), resulting in greater competition and binding by the ADC. However, the actual competition is more complex than the diagram, with avidity dependence on valency, affinity, expression density, linker format, etc^45^. Antibodies with low (monovalent) affinity can still bind with very high avidity, yielding a low “effective” K_D_ on high expressing cells. For example, clone G98A from Tang et al. has a sub-nanomolar effective affinity on high expressing cells (0.5 nM apparent K_D_) despite a low monovalent affinity (270 nM K_D_). This also results in negligible binding to low expressing cells (similar to background)^38^. This high effective affinity (greater than the ADC Kadcyla) and negligible binding to low expressing cells may initially appear promising for a HALA antibody. However, in the presence of a high affinity antibody/ADC, this low affinity agent would rapidly be competed off the cell surface due to antibody ‘wobbling’^46^. A more detailed description of binding and competition on the cell surface is needed to identify effective HALA antibodies.

When cells are exposed to a mixture of HALA and ADC antibodies, the on-rate of binding dominates. Since antibodies have similar on-rates, the initial ratio of HALA antibody versus ADC bound is proportional to the concentration (or dose in vivo). Analogous to multiple reaction systems, the ADC and HALA antibodies are under kinetic control—the binding is determined by the on-rate, which is proportional to the concentration in solution. As time progresses, the higher affinity ADCs begin to outcompete the lower affinity HALA antibodies to approach the thermodynamic equilibrium. Over long times, the antibodies/ADCs would all reach equilibrium, or thermodynamic control, where the higher affinity ADC dominates binding. However, when targeting live cells, this competition occurs for a limited time—until the target is internalized. If the HALA antibody can outcompete the ADC for this time period, it efficiently competes for binding, allowing deeper tissue penetration. If the shift from kinetic control to thermodynamic control happens faster than the rate of internalization, then the ADC outcompetes the HALA antibody. In other words, if the shift from kinetic control to thermodynamic control happens slower than internalization, the antibodies are ‘trapped’ in their kinetic-controlled state. The amount of competition is dictated by Ψ, the ratio between binding competition and internalization (Fig. 2). The goal of this work is to optimize the kinetics such that the HALA antibody outcompetes the ADC on high expression cells but not low expression cells. Ψ is therefore large for high expressing cells and small for low expressing cells.

Efficient competition, as determined by Ψ, is not enough to ensure high ADC payload uptake in all cells, however. The total antibody dose (ADC plus HALA antibody) also needs to be sufficient to overcome local metabolism in the tissue. Therefore, there are two requirements to increase penetration to the center of the spheroid. First, the HALA antibody must be able to compete for binding, as determined by Ψ. Second, the total dose of HALA antibody plus ADC must be sufficient to overcome internalization, and this competition is described by the Thiele modulus^36,47,48^. These two conditions are required to increase penetration to the center of a spheroid (Fig. 3 and Supplemental Fig. S3). The dimensionless numbers are more universal for describing uptake, since the explicit values of affinity and dosing are specific to HER2. However, the dimensional values are more intuitive and therefore shown in Fig. 3.

When the Thiele modulus is close to one, the dose of HALA antibody and ADC has achieved saturation, which overcomes internalization. If the dose of HALA antibody is too high, then the Thiele modulus will be very low, supersaturating the tumor and lowering ADC uptake. To maintain good penetration, Ψ must also be around 10. If the value is much greater than 10, the ADC will penetrate well for high expression tumors. However, the Ψ value will remain high even in tumors with lower target expression, and the HALA antibody will compete too much with the ADC for these lower expression cells.

The Krogh Cylinder model for in vivo simulations adds the additional effects of plasma clearance, blood vessel permeability, and bystander effects (Fig. 4); however, this added complexity does not qualitatively impact how the antibodies distribute within the tissue. The total dose (ADC plus HALA antibody) needs to be sufficient to overcome internalization, and the affinity must be high enough for efficient competition. For simplicity, plasma clearance is assumed to be the same for both antibodies.

The shape of the curves for both T-MMAE and T-DM1 are similar in the Krogh cylinder model. A sufficient dose and HALA antibody competition are needed to facilitate penetration deeper into the tissue. Overall, the bystander effect from T-MMAE shows better distribution into the cells and is able to maintain a minimum concentration for antitumor activity of 150 nM at a wider range of binding affinity and receptor expression^49^. This suggests payloads that exhibit the bystander effect can partially compensate for heterogeneous distribution from either poor ADC tissue penetration or heterogeneous target expression, consistent with clinical experience^50^. However, as seen in other systems, higher ADC tissue penetration is still more effective than relying on bystander effects to overcome tissue penetration limitations^51^.

To optimally design a HALA antibody for use as a carrier dose, the ADC payload uptake in cells distant from tumor vessels can be graphed as a function of HALA antibody affinity and receptor expression (Fig. 6). By selecting an affinity that is high enough to achieve efficient tissue penetration in high expression tumors but low enough not to compete with the ADC under lower expression conditions, optimal in situ competition and maximum efficacy across a range of expression levels can be achieved.

Ultimately, effective ADC treatment requires a positive therapeutic window, where the treatment regimen can achieve significant efficacy relative to toxicity. Therefore, the potential impact of HALA antibodies on toxicity merits discussion. Since HALA antibodies are anticipated to be well-tolerated (similar to typical monoclonal antibodies) they are not expected to increase toxicity by themselves for most targets. Because the toxicity of most ADCs is non-target mediated^52,53^, HALA antibodies are not expected to significantly increase or decrease toxicity. Rather, they are expected to improve the therapeutic window by having a negligible impact on toxicity while significantly improving efficacy. It should be noted, however, that there are cases where target-mediated toxicity is significant (e.g. TROP-2)^54^. In these cases, the HALA antibody is not expected to compete for binding in lower expression tissues. Thus, a higher affinity antibody carrier dose (or lower DAR) may be more appropriate in cases with significant target-mediated toxicity by both lowering uptake in healthy tissue to decrease toxicity and increasing tumor penetration to improve ADC efficacy.

## Conclusion

High Avidity, Low Affinity (HALA) antibodies may serve as optimal carrier doses to increase efficacy of ADCs across a range in cellular expression, even within the same patient or the same tumor. Mechanistic simulations in monolayer, spheroid and Krogh Cylinder models provide insight to the dependency of ADC distribution on coadministered antibody binding affinity, receptor expression, and internalization rate. The data indicate that optimization of the co-administered antibody affinity with ADCs can enable an optimal ratio between competition and internalization to achieve maximum efficacy.

## Supplementary Information

Supplementary Information.

## Data Availability

All data generated or analyzed during this study are included in this published article [and its supplementary information files].

## References

[1] Dean AQ, Luo S, Twomey JD, Zhang B (2021). Targeting cancer with antibody-drug conjugates: Promises and challenges. MAbs.

[2] Khongorzul P, Ling CJ, Khan FU, Ihsan AU, Zhang J (2020). Antibody-drug conjugates: A comprehensive review. Mol. Cancer Res..

[3] Joubert N, Beck A, Dumontet C, Denevault-Sabourin C (2020). Antibody-drug conjugates: The last decade. Pharmaceuticals (Basel).

[4] McKertish CM, Kayser V (2021). Advances and limitations of antibody drug conjugates for cancer. Biomedicines.

[5] Polakis P (2016). Antibody drug conjugates for cancer therapy. Pharmacol. Rev..

[6] Bhatnagar S, Deschenes E, Liao J, Cilliers C, Thurber GM (2014). Multichannel imaging to quantify four classes of pharmacokinetic distribution in tumors. J. Pharm. Sci..

[7] Thurber GM, Schmidt MM, Wittrup KD (2008). Antibody tumor penetration: Transport opposed by systemic and antigen-mediated clearance. Adv. Drug Deliv. Rev..

[8] Rhoden JJ, Wittrup KD (2012). Dose dependence of intratumoral perivascular distribution of monoclonal antibodies. J. Pharm. Sci..

[9] Junutula JR (2008). Site-specific conjugation of a cytotoxic drug to an antibody improves the therapeutic index. Nat. Biotechnol..

[10] Donaghy H (2016). Effects of antibody, drug and linker on the preclinical and clinical toxicities of antibody-drug conjugates. MAbs.

[11] Ponte JF (2021). Antibody Co-administration can improve systemic and local distribution of antibody-drug conjugates to increase. Mol. Cancer Ther..

[12] Yamada K, Yuji I (2019). Recent chemical approaches for site-specific conjugation of native antibodies: Technologies toward next-generation antibody-drug conjugates. ChemBioChem.

[13] Zhou Q (2017). Site-specific antibody conjugation for ADC and beyond. Biomedicines.

[14] Sadiki A (2020). Site-specific conjugation of native antibody. Antib. Ther..

[15] Matsuda Y, Mendelsohn BA (2021). Recent advances in drug-antibody ratio determination of antibody-drug conjugates. Chem. Pharm. Bull..

[16] Behrens CR (2015). Antibody-drug conjugates (ADCs) derived from interchain cysteine cross-linking demonstrate improved homogeneity and other pharmacological properties over conventional heterogeneous ADCs. Mol. Pharm..

[17] Walsh SJ (2020). Site-selective modification strategies in antibody–drug conjugates. Chem. Soc. Rev..

[18] van Geel R (2015). Chemoenzymatic conjugation of toxic payloads to the globally conserved N-glycan of native mAbs provides homogeneous and highly efficacious antibody-drug conjugates. Bioconjug. Chem..

[19] Nessler I (2020). Increased tumor penetration of single-domain antibody-drug conjugates improves. Cancer Res..

[20] Deonarain MP (2018). Small-format drug conjugates: A viable alternative to ADCs for solid tumours?. Antibodies (Basel).

[21] Deonarain MP, Xue Q (2020). Tackling solid tumour therapy with small-format drug conjugates. Antib. Ther..

[22] Wang B, Gallolu Kankanamalage S, Dong J, Liu Y (2021). Optimization of therapeutic antibodies. Antib. Ther..

[23] Annunziata CM (2013). Phase 1, open-label study of MEDI-547 in patients with relapsed or refractory solid tumors. Investig. New Drugs.

[24] Bennett G (2020). MMAE delivery using the. Mol. Cancer Ther..

[25] Lee NK, Su Y, Bidlingmaier S, Liu B (2019). Manipulation of cell-type selective antibody internalization by a guide-effector bispecific design. Mol. Cancer Ther..

[26] Cilliers C, Menezes B, Nessler I, Linderman J, Thurber GM (2018). Improved tumor penetration and single-cell targeting of antibody-drug conjugates increases anticancer efficacy and host survival. Cancer Res..

[27] Singh AP (2020). Antibody coadministration as a strategy to overcome binding-site barrier for ADCs: A quantitative investigation. AAPS J..

[28] Menezes B, Cilliers C, Wessler T, Thurber GM, Linderman JJ (2020). An agent-based systems pharmacology model of the antibody-drug conjugate kadcyla to predict efficacy of different dosing regimens. AAPS J..

[29] Menezes B, Linderman JJ, Thurber GM (2021). Simulating the selection of resistant cells with bystander killing and antibody coadministration in heterogeneous HER2 positive tumors. Drug Metab. Dispos..

[30] Crothers DM, Metzger H (1972). The influence of polyvalency on the binding properties of antibodies. Immunochemistry.

[31] Wittrup KD, Tidor B, Hackel BJ, Sarkar CA (2020). Quantitative Fundamentals of Molecular and Cellular Bioengineering.

[32] Khera E (2021). Quantifying ADC bystander payload penetration with cellular resolution using pharmacodynamic mapping. Neoplasia.

[33] Khera E, Cilliers C, Bhatnagar S, Thurber GM (2018). Computational transport analysis of antibody-drug conjugate bystander effects and payload tumoral distribution: Implications for therapy. Mol. Syst. Des. Eng..

[34] Bartelink IH (2019). Tumor drug penetration measurements could be the neglected piece of the personalized cancer treatment puzzle. Clin. Pharmacol. Ther..

[35] Cilliers C (2016). Modeling of antibody-drug conjugates: Connecting tissue and cellular distribution to whole animal pharmacokinetics and potential implications for efficacy. AAPS J..

[36] Thurber GM, Weissleder R (2011). A systems approach for tumor pharmacokinetics. PLoS ONE.

[37] Jain RK, Baxter LT (1988). Mechanisms of heterogeneous distribution of monoclonal antibodies and other macromolecules in tumors: Significance of elevated interstitial pressure. Cancer Res..

[38] Tang Y (2007). Regulation of antibody-dependent cellular cytotoxicity by IgG intrinsic and apparent affinity for target antigen. J. Immunol..

[39] Drago JZ, Modi S, Chandarlapaty S (2021). Unlocking the potential of antibody-drug conjugates for cancer therapy. Nat. Rev. Clin. Oncol..

[40] Nessler I, Menezes B, Thurber GM (2021). Key metrics to expanding the pipeline of successful antibody-drug conjugates. Trends Pharmacol. Sci..

[41] Nejadmoghaddam MR (2019). Antibody-drug conjugates: Possibilities and challenges. Avicenna J. Med. Biotechnol..

[42] Lucas AT, Moody A, Schorzman AN, Zamboni WC (2021). Importance and considerations of antibody engineering in antibody-drug conjugates development from a clinical pharmacologist's perspective. Antibodies (Basel).

[43] Sun X (2017). Effects of drug-antibody ratio on pharmacokinetics, biodistribution, efficacy, and tolerability of antibody-maytansinoid conjugates. Bioconjug. Chem..

[44] Lu G (2020). Co-administered antibody improves penetration of antibody-dye conjugate into human cancers with implications for antibody-drug conjugates. Nat. Commun..

[45] Csizmar CM (2019). Multivalent ligand binding to cell membrane antigens: Defining the interplay of affinity, valency, and expression density. J. Am. Chem. Soc..

[46] Ong GL, Marria V, Mattes MJ (1994). The fate of antibodies and their radiolabels bound to tumor cells in vitro: The effect of cross-linking at the cell surface and of anti-idiotype antibodies. Cancer Immunol. Immunother..

[47] Hendriks BS, Opresko LK, Wiley HS, Lauffenburger D (2003). Coregulation of epidermal growth factor receptor/human epidermal growth factor receptor 2 (HER2) levels and locations: Quantitative analysis of HER2 overexpression effects. Cancer Res..

[48] Lin J, Sagert J (2018). Innovations for Next-Generation Antibody-Drug Conjugates.

[49] Li F (2017). Tumor-associated macrophages can contribute to antitumor activity through FcγR-mediated processing of antibody-drug conjugates. Mol. Cancer Ther..

[50] Ogitani Y (2016). DS-8201a, A Novel HER2-targeting ADC with a novel DNA topoisomerase I inhibitor, demonstrates a promising antitumor efficacy with differentiation from T-DM1. Clin. Cancer Res..

[51] Khera (2022). Cellular-resolution imaging of bystander payload tissue penetration from antibody-drug conjugates. Mol. Cancer Ther..

[52] Donaghy H (2016). Effects of antibody, drug and linker on the preclinical and clinical toxicities of antibody-drug conjugates. MAbs.

[53] Gorovits B, Krinos-Fiorotti C (2013). Proposed mechanism of off-target toxicity for antibody–drug conjugates driven by mannose receptor uptake. Cancer Immunol. Immunother..

[54] Strop P, Tran TT, Dorywalska M (2015). RN927C, a site-specific trop-2 antibody-drug conjugate (ADC) with enhanced stability, is highly efficacious in preclinical solid tumor models. Mol. Cancer Ther..

