# Supplementary material for: Design of high avidity and low affinity antibodies for in situ control of antibody drug conjugate targeting
Source: Sci Rep. 2022 May 10;12:7677. doi: 10.1038/s41598-022-11648-0 (PMC9090802; doi:10.1038/s41598-022-11648-0)
Supplement: Supplementary file 1 — Supplementary Information. [file 41598_2022_11648_MOESM1_ESM.docx]

**Design of High Avidity and Low Affinity Antibodies for In Situ Control of Antibody Drug Conjugate Targeting**

Reginald Evans and Greg Thurber

1. Differential Equations of Reaction Network, Spheroid and Krogh Cylinder Models

Tables S1 and S2

1. Derivation of Dimensionless Competition Number
2. Derivation of Equilibrium Binding
3. Definition of Thiele Modulus
4. Figures S1 – S8
5. **Differential Equations of Spheroid and Krogh Cylinder Models**

Spheroid competition model

$\frac{d\left[ Ab_{ADC} \right]}{dt}=D_{eff}(\frac{1}{r^{2}}\frac{d}{dr}\left( r^{2}\frac{d\left[ Ab_{ADC} \right]}{dr} \right))-{2k}_{on\left( 1ADC \right)}\left[ Ag \right]\left[ Ab_{ADC} \right]+k_{off\left( 1ADC \right)}\left[ M_{ADC} \right]$ 1.

$\frac{d\left[ Ab_{HALA} \right]}{dt}=D_{eff}(\frac{1}{r^{2}}\frac{d}{dr}\left( r^{2}\frac{d\left[ Ab_{HALA} \right]}{dr} \right))-{2k}_{on\left( 1HALA \right)}\left[ Ag \right]\left[ Ab_{HALA} \right]+k_{off\left( 1HALA \right)}\left[ M_{HALA} \right]$ 2.

Monovalent Binding event

$\frac{d\left[ M_{ADC} \right]}{dt}={2k}_{on\left( 1ADC \right)}\left[ Ag \right]\left[ Ab_{ADC} \right]-k_{off\left( 1ADC \right)}\left[ M_{ADC} \right]-k_{on\left( 2ADC \right)}\left[ M_{ADC} \right]\left[ Ag \right]+{2k}_{off\left( 2ADC \right)}\left[ B_{ADC} \right]-k_{e}[M_{ADC}]$ 3.

$\frac{d\left[ M_{HALA} \right]}{dt}={2k}_{on\left( 1HALA \right)}\left[ Ag \right]\left[ Ab_{HALA} \right]-k_{off\left( 1HALA \right)}\left[ M_{HALA} \right]-k_{on\left( 2HALA \right)}\left[ M_{HALA} \right]\left[ Ag \right]+{2k}_{off\left( 2HALA \right)}[B_{HALA}]-k_{e}[M_{HALA}]$ 4.

Bivalent Binding event

$\frac{d\left[ B_{ADC} \right]}{dt}=k_{on\left( 2ADC \right)}\left[ M_{ADC} \right]\left[ Ag \right]-{2k}_{off\left( 2ADC \right)}[B_{ADC}]-k_{e}[B_{ADC}]$ 5.

$\frac{d\left[ B_{HALA} \right]}{dt}=k_{on\left( 2HALA \right)}\left[ M_{HALA} \right]\left[ Ag \right]-{2k}_{off(2HALA)}[B_{HALA}]-k_{e}[B_{ADC}]$ 6.

Antigen Balance

$\frac{d\left[ Ag \right]}{dt}=-{2k}_{on\left( 1ADC \right)}\left[ Ag \right]\left[ Ab_{ADC} \right]+k_{off\left( 1ADC \right)}\left[ M_{ADC} \right]- {2k}_{on\left( 1HALA \right)}\left[ Ag \right]\left[ Ab_{HALA} \right]+k_{off\left( 1HALA \right)}\left[ M_{HALA} \right]- k_{on\left( 2ADC \right)}\left[ M_{ADC} \right]\left[ Ag \right]-k_{on\left( 2HALA \right)}\left[ M_{HALA} \right]\left[ Ag \right]+{2k}_{off\left( 2ADC \right)}\left[ B_{ADC} \right]+{2k}_{off\left( 2HALA \right)}\left[ B_{HALA} \right]+R_{s}-k_{e}[Ag]$ 7.

Receptor Synthesis Rate for Steady State Surface Concentration

$R_{s}=k_{e}{[Ag]}_{0}$

Initial Conditions at t = 0

$$\left[ Ab_{HALA} \right]=[Ab_{HALA}]_{0}$$

$$\left[ Ab_{ADC} \right]=[Ab_{ADC}]_{0}$$

[Ag] = [Ag]_0_

$$\left[ M_{HALA} \right]=0$$

$$\left[ M_{ADC} \right]=0$$

$$\left[ B_{HALA} \right]=0$$

$$\left[ B_{ADC} \right]=0$$

Boundary Conditions

r=R_spheroid edge,_ $\left[ Ab_{HALA} \right]=[Ab_{HALA}]_{0}$

r=R_spheroid edge,_ $\left[ Ab_{ADC} \right]=[Ab_{ADC}]_{0}$

r=R_spheroid center_ $D_{eff}\left( \frac{d\left[ Ab_{HALA} \right]}{dr} \right)=0$

r=R_spheroid center_ $D_{eff}\left( \frac{d\left[ Ab_{HALA} \right]}{dr} \right)=0$

Krogh Cylinder

$\frac{d\left[ HALA \right]}{dt}=D_{eff}\left( \frac{1}{r}\frac{d}{dr}\left( r\frac{d\left[ HALA \right]}{dr} \right) \right)-{2k}_{on1\left( HALA \right)}\left[ Ag \right]*\frac{\left[ HALA \right]}{\varepsilon}+k_{off\left( 1HALA \right)}\left[ M_{HALA} \right]$ 1.

$\frac{d\left[ ADC \right]}{dt}=D_{eff}(\frac{1}{r}\frac{d}{dr}\left( r\frac{d\left[ ADC \right]}{dr} \right))-{2k}_{on1\left( ADC \right)}\left[ Ag \right]*\frac{\left[ ADC \right]}{\varepsilon}+k_{off\left( ADC \right)}\left[ M_{ADC} \right]$ 2.

Monovalent Binding event

$\frac{d\left[ M_{HALA} \right]}{dt}={2k}_{on\left( 1HALA \right)}\left[ Ag \right]*\frac{\left[ HALA \right]}{\varepsilon}-k_{off\left( 1HALA \right)}\left[ M_{HALA} \right]-k_{on\left( 2HALA \right)}\left[ M_{HALA} \right]\left[ Ag \right]+{2k}_{off\left( 2HALA \right)}\left[ B_{HALA} \right]-k_{e}[M_{HALA}]$ 3.

$\frac{d\left[ M_{ADC} \right]}{dt}={2k}_{on\left( 1ADC \right)}\left[ Ag \right]*\frac{\left[ ADC \right]}{\varepsilon}-k_{off\left( 1ADC \right)}\left[ M_{ADC} \right]-k_{on\left( 2ADC \right)}\left[ M_{ADC} \right]\left[ Ag \right]+{2k}_{off\left( 2ADC \right)}[B_{ADC}]-k_{e}[M_{ADC}]$ 4.

Bivalent Binding event

$\frac{d\left[ B_{HALA} \right]}{dt}=k_{on\left( 2HALA \right)}\left[ M_{HALA} \right]\left[ Ag \right]-{2k}_{off\left( 2HALA \right)}[B_{HALA}]-k_{e}[B_{HALA}]$ 5.

$\frac{d\left[ B_{ADC} \right]}{dt}=k_{on\left( 2ADC \right)}\left[ M_{ADC} \right]\left[ Ag \right]-{2k}_{off(2ADC)}[B_{ADC}]-k_{e}[B_{ADC}]$ 6.

Internalized HALA/ADC

$\frac{d\left[ C_{int,Hala} \right]}{dt}=k_{e}\left[ M_{HALA} \right]+k_{e}\left[ B_{HALA} \right]-k_{deg}[C_{int,MHALA}]-k_{deg}[C_{int,BHALA}]$ 7.

$\frac{d\left[ C_{int,ADC} \right]}{dt}=k_{e}\left[ M_{ADC} \right]+k_{e}\left[ B_{ADC} \right]-k_{deg}[C_{int,MADC}]-k_{deg}[C_{int,BADC}]$ 8.

Extracellular payload

$\frac{d\left[ C{ext}_{P} \right]}{dt}=D_{eff}\left( \frac{1}{r}\frac{d}{dr}\left( r\frac{d\left[ C_{ext,P} \right]}{dr} \right) \right)-k_{in\left( P \right)}\frac{1-\varepsilon\left( p \right)}{\varepsilon\left( p \right)}{[C}_{ext,P}]+k_{out,P}[C_{int,P}]$ 9.

Intracellular Payload

$\frac{d\left[ C{int}_{P} \right]}{dt}=k_{in\left( P \right)}\frac{1-\varepsilon(p)}{\varepsilon(p)}{[C}_{ext,P}]-k_{out,P}[C_{int,P}]- \frac{k_{on,P}}{\left( 1-\varepsilon\left( p \right) \right)R} \left( P_{target}C_{bound,P} \right)C_{int,P}+k_{off,P}C_{bound,P}+ k_{in,P}C_{lyso,P}$ 10.

Bound payload

$\frac{d\left[ C{bound}_{P} \right]}{dt}=\frac{k_{on,P}}{\left( 1-\varepsilon\left( p \right) \right)R} \left( P_{target}C_{bound,P} \right)C_{int,P}-k_{off,P}C_{bound,P}$ 11.

Lysosome payload

$\frac{d\left[ C{int}_{P} \right]}{dt}=DAR*[C_{int,ADC}]-k_{in,P}C_{lyso,P}$ 12.

Antigen Balance

$\frac{d\left[ Ag \right]}{dt}=-{2k}_{on\left( 1HALA \right)}\left[ Ag \right]\left[ HALA \right]+k_{off\left( 1HALA \right)}\left[ M_{HALA} \right]- {2k}_{on\left( 1ADC \right)}\left[ Ag \right]\left[ ADC \right]+k_{off\left( 1b \right)}\left[ M_{ADC} \right]- k_{on\left( 2HALA \right)}\left[ M_{HALA} \right]\left[ Ag \right]-k_{on\left( 2ADC \right)}\left[ M_{ADC} \right]\left[ Ag \right]+{2k}_{off\left( 2HALA \right)}\left[ B_{HALA} \right]+{2k}_{off\left( 2ADC \right)}\left[ B_{ADC} \right]+R_{s}-k_{e}[Ag]$ 13.

Receptor Synthesis Rate for Steady State Surface Concentration

$$R_{s}=k_{e}{[Ag]}_{0}$$

Initial Conditions at t = 0

$$\left[ Ab_{HALA} \right]=[Ab_{HALA}]_{0}$$

$$\left[ Ab_{ADC} \right]=[Ab_{ADC}]_{0}$$

[Ag] = [Ag]_0_

$$\left[ M_{HALA} \right]=0$$

$$\left[ M_{ADC} \right]=0$$

$$\left[ B_{HALA} \right]=0$$

$$\left[ B_{ADC} \right]=0$$

Boundary Conditions (ADC, HALA, payload)

${-D}_{eff}{\frac{dC_{free}}{dr}|}_{r=R_{capillary}} = P\left( C_{plasma,ADC/HALA}-\frac{C_{free}}{\varepsilon} \right)$ 1.

$D_{eff}{\frac{dC_{free}}{dr}|}_{r=R_{Krogh}} = 0$ 2.

${-D}_{eff}{\frac{dC_{ext,P}}{dr}|}_{r=R_{capillary}} = P_{p}\left( C_{plasma,P}-\frac{C_{ext,P}}{\varepsilon_{p}(1+R)} \right)$ 3.

$D_{eff}{\frac{dC_{ext,P}}{dr}|}_{r=R_{Krogh}} = 0$ 4.

$C_{plasma, ADC/HALA}=[C]_{0}*(A*\exp\left( -k_{\alpha}*t \right)+\left( 1-A \right)* \exp\left( -k_{b}*t \right))$ 5.

$C_{plasma, P}=0$ 6.

| Parameter | Value | Unit | Description | Reference |
| --- | --- | --- | --- | --- |
| Parameters used in all 3 Models | | | | |
| k_on_,_ADC/HALA_ | 10^5^ | M^-1^s^-1^ | ADC or HALA monovalent binding rate | Estimated from 1,2 |
| K_d_,_ADC_ | 5 | nM | ADC dissociation constant | 3 |
| K_d_,_HALA_ | 0.1-10,000 ^a^  1-10,000 ^b,c^ | nM | HALA dissociation constant | Varies |
| k_off_,_ADC/HALA_ | = k_on_,_ADC/HALA*_K_d_,_ADC/HALA_ | s^-1^ | ADC or HALA dissociation rate | Calculated |
| k_on2_,_ADC/HALA_ | 3.36x10^7^ | M^-1^s^-1^ | ADC or HALA bivalent binding rate | 4 |
| k_off2_,_ADC/HALA_ | k_off_,_ADC/HALA_ | s^-1^ | ADC or HALA bivalent dissociation rate | Identical to k_off_,_ADC/HALA_ |
| Ag_0_ | 1x10^3^ – 1x10^11 a^  833 ^b,c^ | Receptors Cell^-1^  nM | Initial antigen concentration | 5^a^  Calculated ^b,c^ |
| k_e_ | 0.01- 1x10^7 a^  3.3x10^5 b,c^ | s^-1^ | ADC/HALA net internalization rate | Varies  6,7,8,9 |
| Dose_ADC_ | 0.1-1000 ^a^  5 ^b,c^ | nM | Initial Concentration of ADC | Varies  Calculated |
| Dose_HALA_ | 0.001- 100,000 ^a^  1-1000^b,c^ | nM | Initial Concentration of HALA | Varies |
| Parameters used in Spheroid and/or Krogh Cylinder Model | | | | |
| D_e_ | 10 ^b,c^ | μm^2^/s | Diffusivity of ADC/HALA | 3 |
| ε | 0.2 | ND | Tumor void Fraction | 10 |
| R_Spheroid_ | 200^b^ | μm | Spheroid Radius | 11 |
| Parameters used in the Krogh Cylinder Model | | | | |
| R_Krogh_ | 75^c^ | μm | Krogh Cylinder (tumor radius) | 12 |
| R_Capillary_ | 8^c^ | μm | Capillary Radius | 13 |
| A | 0.43 | ND | Fraction of Alpha Clearance | 3 |
| k_α_ | 0.0866 | h^-1^ | Alpha phase clearance rate | 14 |
| k_β_ | 0.0347 | h^-1^ | Beta phase clearance rate | 14 |
| P_ADC_ | 3x10^-9^ | ms^-1^ | Vascular Permeability of ADC/HALA | 15 |
| Q | 0.0015 | mLg^-1^s^-1^ | Tumor Blood  Flow Rate | 16 |
| H | 0.45 | ND | Hematocrit | 17 |
| k_deg_ | 8x10^-6^ | s^-1^ | ADC lysosomal degradation rate | 18 |
| DAR | 4 | ND | Drug to Antibody Ratio | Measured |
| k_in,P_ (DM1) | 5.95x10^-5^ | s^-1^ | Payload influx rate | 6 |
| k_in,P_ (MMAE) | 1.41x10^-3^ | s^-1^ | Payload influx rate | 6 |
| k_out,P_ (DM1) | 3.94x10^-5^ | s^-1^ | Payload efflux rate | 6 |
| k_out,P_ (MMAE) | 6.87x10^-4^ | s^-1^ | Payload efflux rate | 6 |
| D_p_ (DM1) | 9.8 | μm^2^s^-1^ | Payload diffusion coefficient | 19 |
| D_p_ (MMAE) | 14.8 | μm^2^s^-1^ | Payload diffusion coefficient | 19,20 |
| P_P_ | 10^-6^ | m s^-1^ | Free payload vascular permeability | 3 |
| k_on,P_ | 8333 | M^-1^s^-1^ | Payload binding rate | 21 |
| k_off,P_ | 0.003 | s^-1^ | Payload dissociation rate | 21 |
| ε_p_ | 0.44 | ND | Cell void fraction | 22,23 |
| P_target_ | 20 | μM | Microtubule concentration | 21 |

Table S1: Constants used in the models

A = PSI simulations B= Spheroid simulations C= Krogh Cylinder Simulations

ND = non-dimensional

| Variables used in all 3 models | | |
| --- | --- | --- |
| Variable | Unit | Description |
| Ab_ADC_ | nM | Free ADC |
| Ab_HALA_ | nM | Free HALA antibody |
| Ag | nM | Antigen |
| M_HALA_ | nM | Monovalently bound HALA antibody |
| M_ADC_ | nM | Monovalently bound ADC |
| B_ADC_ | nM | Bivalently bound ADC |
| B_HALA_ | nM | Bivalently bound HALA |
| Variables exclusively used in Krogh Cylinder model | | |
| C_int,HALA_ | nM | Internalized Total HALA |
| C_int,MHALA_ | nM | Internalized monovalently bound HALA |
| C_int,BHALA_ | nM | Internalized bivalently bound HALA |
| C_int,ADC_ | nM | Internalized Total ADC |
| C_int,MADC_ | nM | Internalized Monovalent ADC |
| C_int,BADC_ | nM | Internalized Bivalent ADC |
| C_ext,P_ | nM | Extracellular Payload |
| C_int,P_ | nM | Intracellular Payload |
| C_bound,P_ | nM | Bound Payload |
| C_lyso,P_ | nM | Lysosomal Payload |

Table S2: Variables used in the models

1. **Derivation of Dimensionless Competition Number**

Strategy and Description

To generate a simplified description of the competition between a bivalent HALA antibody and a bivalent ADC, several simplifying assumptions were made. First, it is assumed that the ADC is high affinity and has effectively irreversible binding. In practice, this means the ADC is internalized before it has time to dissociate. Second, it is assumed that the concentration of the HALA antibody is much greater than the ADC such that the initial fraction bound is much greater for the HALA antibody, and initial bound ADC is ignored.

Following these assumptions, the initial ‘pool’ of antigen available for binding by the HALA and ADC is slowly depleted over time as more ADC irreversibly binds the antigen, depleting the available antigen 'pool.’ It is assumed that the molecular binding events from the HALA antibody are faster than the binding of the ADC, thereby allowing an equilibrium approximation. Once the equilibrium approximation is made, the rate of ‘depletion’ of the available antigen can be calculated by the binding rate between unoccupied receptor and ADC. This yields the rate of competition by the ADC.

If internalization is not present, the ADC will compete with the HALA antibody indefinitely until it completely occupies all binding sites (due to the irreversible binding assumption). However, the antigen is also being internalized during this time. If the HALA antibody can block binding of the ADC to the target long enough that the target internalizes, the ADC will never be able to bind. Therefore, the overall effective competition of the HALA antibody is the ratio of the rate of ADC binding to the target (in the presence of the HALA antibody) to the internalization rate. If the affinity, concentration, etc. are sufficient, the HALA antibody will be able to prevent binding of the ADC prior to internalization, and the HALA will be an effective competitor. If the ADC can bind faster than the antigen is internalized, then the HALA antibody will not be able to compete for binding with the ADC.

The mathematical derivation of this strategy is shown below:

M = monovalently bound HALA

B = bivalently bound HALA

Ag = antigen

Ab_HALA_ = HALA antibody concentration

ADC = ADC concentration

F = ratio of HALA to ADC concentration

[Ag]_PSSH_ = The concentration of available antigen for binding by HALA and ADC using the Pseudo-Steady State Hypothesis. This is the sum of Ag, M, and 2B that is depleted by ADC binding.

Equilibrium Constant Definitions:

$K_{d,1,HALA}=\frac{k_{off,HALA}}{k_{on,HALA}}=\frac{2\left[ Ag \right][Ab_{HALA}]}{[M_{HALA}]}$ $K_{d,2,HALA}=\frac{k_{off,HALA}}{k_{on,2HALA}}=\frac{\left[ M_{HALA} \right][Ag]}{2[B_{HALA}]}$

Define the kinetic rate of depletion of the available antigen due to binding:

$$\left. \frac{d{[Ag]}_{Pssh}}{dt} \right|_{binding}=-2k_{on,ADC}*[ADC][Ag]$$

$\left. \frac{d{[Ag]}_{Pssh}}{dt} \right|_{binding}=-2k_{on,ADC}*\frac{{[Ab]}_{HALA}}{F}[Ag]$

Assume equilibrium binding of HALA antibody:

$[Ag]_{o,pssh}= \left[ Ag \right]+\left[ M_{HALA} \right]+2\left[ B_{HALA} \right]$

Using Definition of $K_{d1HALA}$ and $K_{d2HALA}$ substitute for ${[M}_{HALA}]$and ${[B}_{HALA}]$

$[Ag]_{o,pssh}=\left[ Ag \right]+\frac{2\left[ Ag \right]\left[ Ab_{HALA} \right]}{{K_{d1}}_{HALA}}+ \frac{2*\left[ M_{HALA} \right][Ag]}{2* {K_{d1}}_{HALA}}$

= $\left[ Ag \right]+\frac{2\left[ Ag \right]\left[ Ab_{HALA} \right]}{{K_{d1}}_{HALA}}+ \frac{\left[ Ab_{HALA} \right][Ag]^{2}}{{K_{d1}}_{HALA}* {K_{d2}}_{HALA}}$

$[Ag]_{o,pssh}=\left[ Ag \right](1+\frac{2*\left[ Ab_{HALA} \right]}{{K_{d1}}_{HALA}}\left( 1+ \frac{\left[ Ag \right]}{{K_{d2}}_{HALA}} \right))$

If [Ag]/K_d,2,HALA_ <<1, then there isn’t any benefit from avidity, so assume >> 1

$[Ag]_{o,pssh}=\left[ Ag \right](1+\frac{2*\left[ Ab_{HALA} \right]}{{K_{d1}}_{HALA}}\left( \frac{\left[ Ag \right]}{{K_{d2}}_{HALA}} \right))$

It is also assumed that a large fraction of the Ag is bound (to provide adequate competition), so it is assumed the blue terms are >>1. Simplifying and solving for Ag:

$[Ag]=\sqrt{\frac{[Ag]_{o,pssh}* {K_{d1}}_{HALA}*{K_{d2}}_{HALA}}{2*\left[ Ab_{HALA} \right]}}$

The free antigen concentration is entered into the kinetic equation for total available Ag: $\left. \frac{d{[Ag]}_{Pssh}}{dt} \right|_{binding}=-2k_{on,ADC}*\frac{{[Ab]}_{HALA}}{F}[Ag]$

$\left. \frac{d{[Ag]}_{Pssh}}{dt} \right|_{binding}=-2k_{on,ADC}*\frac{{[Ab]}_{HALA}}{F}*\sqrt{\frac{[Ag]_{o,pssh}* {K_{d1}}_{HALA}*{K_{d2}}_{HALA}}{2*\left[ Ab_{HALA} \right]}}$

Simplifying:

$=-2k_{on,ADC}*\frac{{[Ab]}_{HALA}}{F}\sqrt{\frac{[Ag]_{o,pssh}* {K_{d1}}_{HALA}*{K_{d2}}_{HALA}}{2*\left[ Ab_{HALA} \right]}}\left( \frac{{K_{d1}}_{HALA}}{{K_{d1}}_{HALA}} \right)$

Rearrange and simplify:

$=-2k_{on,ADC}*\frac{k_{off,HALA}}{{F*kon,}_{HALA}}\sqrt{\frac{[Ag]_{o,pssh}*\left[ Ab_{HALA} \right]*{K_{d2}}_{HALA}}{2*{K_{d1}}_{HALA}}}$

Assume k_on_ are approximately the same for ADC and HALA and cancel:

$\left. \frac{d{[Ag]}_{Pssh}}{dt} \right|_{binding}=-\frac{2k_{off,HALA}}{F}\sqrt{\frac{[Ag]_{o,pssh}*\left[ Ab_{HALA} \right]*{K_{d2}}_{HALA}}{2*{K_{d1}}_{HALA}}}$

As the antigen is being bound by the ADC, there is a parallel competing ‘reaction’ of internalization:

$\left. \frac{d{[Ag]}_{Pssh}}{dt} \right|_{internalization}=-ke*[Ag]_{o,pssh}$

The ratio of these two competing reactions for $[Ag]_{o,pssh}$, one yielding binding by the ADC and the other yielding effective competition, results in the competition number:

$\psi\equiv\frac{-ke*[Ag]_{o,pssh}}{-\frac{2k_{off,HALA}}{F}\sqrt{\frac{[Ag]_{o,pssh}*\left[ Ab_{HALA} \right]*{K_{d2}}_{HALA}}{2*{K_{d1}}_{HALA}}}}$

Simplifying:

$\psi=\frac{ke*F}{2k_{off,HALA}}\sqrt{\frac{2*{K_{d1}}_{HALA}}{\left[ Ab_{HALA} \right]}}\sqrt{\frac{[Ag]_{o,pssh}}{{K_{d2}}_{HALA}}}$

1. **Derivation of Equilibrium Binding**

Derivation of simplified expression for thermodynamic equilibrium with ADC/ HALA binding on a monolayer without internalization

${K_{d}}_{1HALA}=\frac{k_{off,HALA}}{k_{on,HALA}}=\frac{2\left[ Ag \right][Ab_{HALA}]}{[M_{HALA}]}$ ${K_{d}}_{2HALA}=\frac{k_{off,HALA}}{k_{on,2HALA}}=\frac{\left[ M_{HALA} \right][Ag]}{2[B_{HALA}]}$

${K_{d}}_{1ADC}=\frac{k_{off,ADC}}{k_{on,ADC}}=\frac{2\left[ Ag \right][Ab_{ADC}]}{[M_{ADC}]}$ ${K_{d}}_{2ADC}=\frac{k_{off,ADC}}{k_{on,2ADC}}=\frac{\left[ M_{ADC} \right][Ag]}{2[B_{ADC}]}$

Assume $Ab_{HALA} and Ab_{ADC}$are constant (excess antibody). Use conservation of total antigen, assume equilibrium, and enter values from relationships above:

$$[Ag]_{0}=\left[ Ag \right]+ \left[ M_{HALA} \right]+ \left[ M_{ADC} \right]+2\left[ B_{HALA} \right]+2[B_{ADC}]$$

$$[Ag]_{0}=\left[ Ag \right]+ \frac{2\left[ Ag \right][Ab_{HALA}]}{[{K_{d}}_{1HALA}]}+ \frac{2\left[ Ag \right][Ab_{ADC}]}{[{K_{d}}_{1ADC}]}+\frac{2\left[ M_{HALA} \right][Ag]}{2[{K_{d}}_{2HALA}]}+\frac{2\left[ M_{ADC} \right][Ag]}{2[{K_{d}}_{2ADC}]}$$

Factor out [Ag]:

$$[Ag]_{0}=\left[ Ag \right]*[ 1+ \frac{2\left[ Ab_{HALA} \right]}{\left[ {K_{d}}_{1HALA} \right]}+ \frac{2\left[ Ab_{ADC} \right]}{\left[ {K_{d}}_{1ADC} \right]}+\frac{\left[ M_{HALA} \right]}{\left[ {K_{d}}_{2HALA} \right]}+\frac{\left[ M_{ADC} \right]}{\left[ {K_{d}}_{2ADC} \right]}]$$

Substitute approximations for M_HALA_ and M_ADC_ again:

$$[Ag]_{0}=\left[ Ag \right]*[ 1+ \frac{2\left[ Ab_{HALA} \right]}{\left[ {K_{d}}_{1HALA} \right]}+ \frac{2\left[ Ab_{ADC} \right]}{\left[ {K_{d}}_{1ADC} \right]}+\frac{2\left[ Ag \right][Ab_{HALA}]}{[{K_{d}}_{1HALA}][{K_{d}}_{2HALA}]}+\frac{2\left[ Ag \right][Ab_{ADC}]}{[{K_{d}}_{1ADC}][{K_{d}}_{2ADC}]}]$$

Factor out [Ag]

$$[Ag]_{0}=\left[ Ag \right]*[ 1+ \frac{2\left[ Ab_{HALA} \right]}{\left[ {K_{d}}_{1HALA} \right]}+ \frac{2\left[ Ab_{ADC} \right]}{\left[ {K_{d}}_{1ADC} \right]}]+[Ag]^{2}*[\frac{2\left[ Ab_{HALA} \right]}{\left[ {K_{d}}_{1HALA} \right]\left[ {K_{d}}_{2HALA} \right]}$$

$$+\frac{2[Ab_{ADC}]}{[{K_{d}}_{1ADC}][{K_{d}}_{2ADC}]}]$$

At equilibrium, the avidity of antibodies results in mostly bivalent binding. Likewise, it is assumed the antibodies are high affinity where the free antigen concentration is low. Therefore, the free antigen and monovalently bound terms are negligible:

$$[Ag]_{0}=[Ag]^{2}*[\frac{2[Ab_{HALA}]}{[{K_{d}}_{1HALA}][{K_{d}}_{2HALA}]}+\frac{2[Ab_{ADC}]}{[{K_{d}}_{1ADC}][{K_{d}}_{2ADC}]}]$$

$$[Ag]^{2}=\frac{(\frac{[Ag]_{0}}{2})}{\frac{[Ab_{HALA}]}{[{K_{d}}_{1HALA}][{K_{d}}_{2HALA}]}+\frac{[Ab_{ADC}]}{[{K_{d}}_{1ADC}][{K_{d}}_{2ADC}]}}$$

At equilibrium, the avidity of antibodies results in mostly bivalent binding, so the concentration of bivalent antibody at equilibrium is:

$\frac{\left[ M_{HALA} \right]+[B_{HALA}]}{[Ag]_{0}}\approx\frac{[B_{HALA}]}{[Ag]_{0}}$

From definitions of equilibrium:

$[M_{ADC}]=\frac{2\left[ Ag \right][Ab_{ADC}]}{{K_{d}}_{1ADC}}$ $[B_{ADC}]=\frac{\left[ M_{ADC} \right][Ag]}{2*{K_{d}}_{2ADC}}$

Substitute M_HALA_ into B_HALA_:

$$[B_{ADC}]=\frac{2\left[ Ag \right][Ag][Ab_{ADC}]}{2*{K_{d}}_{1ADC}*{K_{d}}_{2ADC}}$$

$$[B_{ADC}]=\frac{[Ag]^{2}[Ab_{ADC}]}{{K_{d}}_{1ADC}*{K_{d}}_{2ADC}}$$

Substitute $[Ag]^{2}$ into equation:

$$\left[ B_{ADC} \right]=\frac{\left[ Ab_{ADC} \right]}{{K_{d}}_{1ADC}*{K_{d}}_{2ADC}}* \frac{(\frac{[Ag]_{0}}{2})}{\frac{[Ab_{ADC}]}{{K_{d}}_{1ADC}*{K_{d}}_{2ADC}}+\frac{[Ab_{HALA}]}{{K_{d}}_{1HALA}*{K_{d}}_{2HALA}}}$$

Solve for B_ADC_

$$\frac{\left[ B_{ADC} \right]}{\left( \frac{[Ag]_{0}}{2} \right)}= \frac{\frac{\left[ Ab_{ADC} \right]}{{K_{d}}_{1ADC}*{K_{d}}_{2ADC}}}{\frac{[Ab_{ADC}]}{{K_{d}}_{1ADC}*{K_{d}}_{2ADC}}+\frac{[Ab_{HALA}]}{{K_{d}}_{1HALA}*{K_{d}}_{2HALA}}}$$

$$\frac{\left[ B_{ADC} \right]}{\left( \frac{[Ag]_{0}}{2} \right)}= \frac{\left[ Ab_{ADC} \right]}{\left[ Ab_{ADC} \right]+[Ab_{HALA}]\frac{{K_{d}}_{1ADC}*{K_{d}}_{2ADC}}{{K_{d}}_{1HALA}*{K_{d}}_{2HALA}}}$$

To simplify the ratio of monovalent and bivalent equilibrium binding, an avidity model is used. In this model, the off-rate is assumed constant, and the effective on-rate is a product of the intrinsic binding rate and local concentration:

k_off,1_ = k_off,2_

$$\frac{{K_{d}}_{2ADC}}{{K_{d}}_{2HALA}}=\frac{\frac{k_{off,ADC}}{k_{on,2ADC}}}{\frac{k_{off,HALA}}{k_{on,2HALA}}}$$

Definition of $k_{on,2}=\frac{3}{8\pi r^{2}cellr_{igG}N_{a}}$ Reference [4]

Substitute definition into previous equation

$$\frac{{K_{d}}_{2ADC}}{{K_{d}}_{2HALA}}=\frac{\frac{k_{off,ADC}}{k_{on,ADC}* \frac{3}{8\pi r^{2}cellr_{igG}N_{a}}}}{\frac{k_{off,HALA}}{k_{on,HALA}* \frac{3}{8\pi r^{2}cellr_{igG}N_{a}}}}$$

Assume $r_{cell} and r_{IgG}$ are equal between ADC and HALA antibody:

$$\frac{{K_{d}}_{1ADC}}{{K_{d}}_{1HALA}}= \frac{{K_{d}}_{2ADC}}{{K_{d}}_{2HALA}}$$

Plug in definition into B_ADC_ equation:

$$\frac{\left[ B_{ADC} \right]}{\left( \frac{[Ag]_{0}}{2} \right)}= \frac{\left[ Ab_{ADC} \right]}{\left[ Ab_{ADC} \right]+[Ab_{HALA}]\left( \frac{{K_{d}}_{1ADC}}{{K_{d}}_{1HALA}} \right)^{2}}$$

Factor out Ab_ADC_

Assume ${K_{d}}_{ADC}< {K_{d}}_{HALA}$

Assume $[Ab_{ADC}]< [Ab_{HALA}]$

$$\frac{\left[ B_{ADC} \right]}{\left( \frac{[Ag]_{0}}{2} \right)}= \frac{1}{1+\frac{[Ab_{HALA}]}{\left[ Ab_{ADC} \right]}\left( \frac{{K_{d}}_{1ADC}}{{K_{d}}_{1HALA}} \right)^{2}}$$

F = $\frac{[{Ab}_{HALA}]}{[{Ab}_{ADC}]}$

$$\frac{\left[ B_{ADC} \right]}{\left( \frac{[Ag]_{0}}{2} \right)}= \frac{1}{1+F\left( \frac{{K_{d}}_{1ADC}}{{K_{d}}_{1HALA}} \right)^{2}}$$

1. **Definition of Thiele Modulus**

Φ^2^ is previously defined as follows from previous literature [7]

Φ^2^ =$\frac{k_{e}*\left[ Ag \right]*(R_{spheroid}^{2})}{\varepsilon*D*Ab}$

R spheroid is the characteristic length of the spheroid which is R/3 thus Φ^2^ becomes

Φ^2^ =$\frac{k_{e}*\left[ Ag \right]*(R_{spheroid}^{2})}{\varepsilon*D*Ab*9}$

In the Krogh Cylinder model Φ^2^ is defined as

Φ^2^ =$\frac{k_{e}*\left[ Ag \right]*(R_{Krogh\_cylinder}^{2})}{2P*R_{Capillary}*Ab}$

| Variable | Description |
| --- | --- |
| k_e_ | Internalization rate |
| Ag | Total cell receptors |
| R_spheroid_ | Spheroid Radius |
| R_Krogh_Cylinder_ | Krogh Cylinder Radius |
| D | Diffusion coefficient |
| Ab | Total Antibody Dose |
| Ε | Tumor Void fraction |
| P | Permeability |
| R_Capillary_ | Capillary Radius |

1. **Figures**


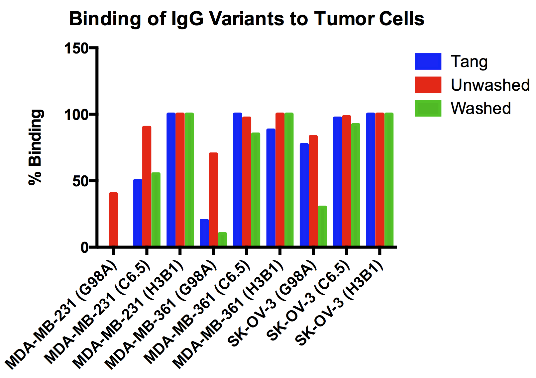


**Figure S1: Validation of monolayer simulations.** Validation of monolayer simulations of different antibody clones at different cell expression. Binding assays results were previously published [5]. “Unwashed” simulations modeled the association phase of competition binding only, which accounts for the higher % of binding. The “washed” simulations accounts for the washing step of blocking studies, to include dissociation which has better congruence with published results.


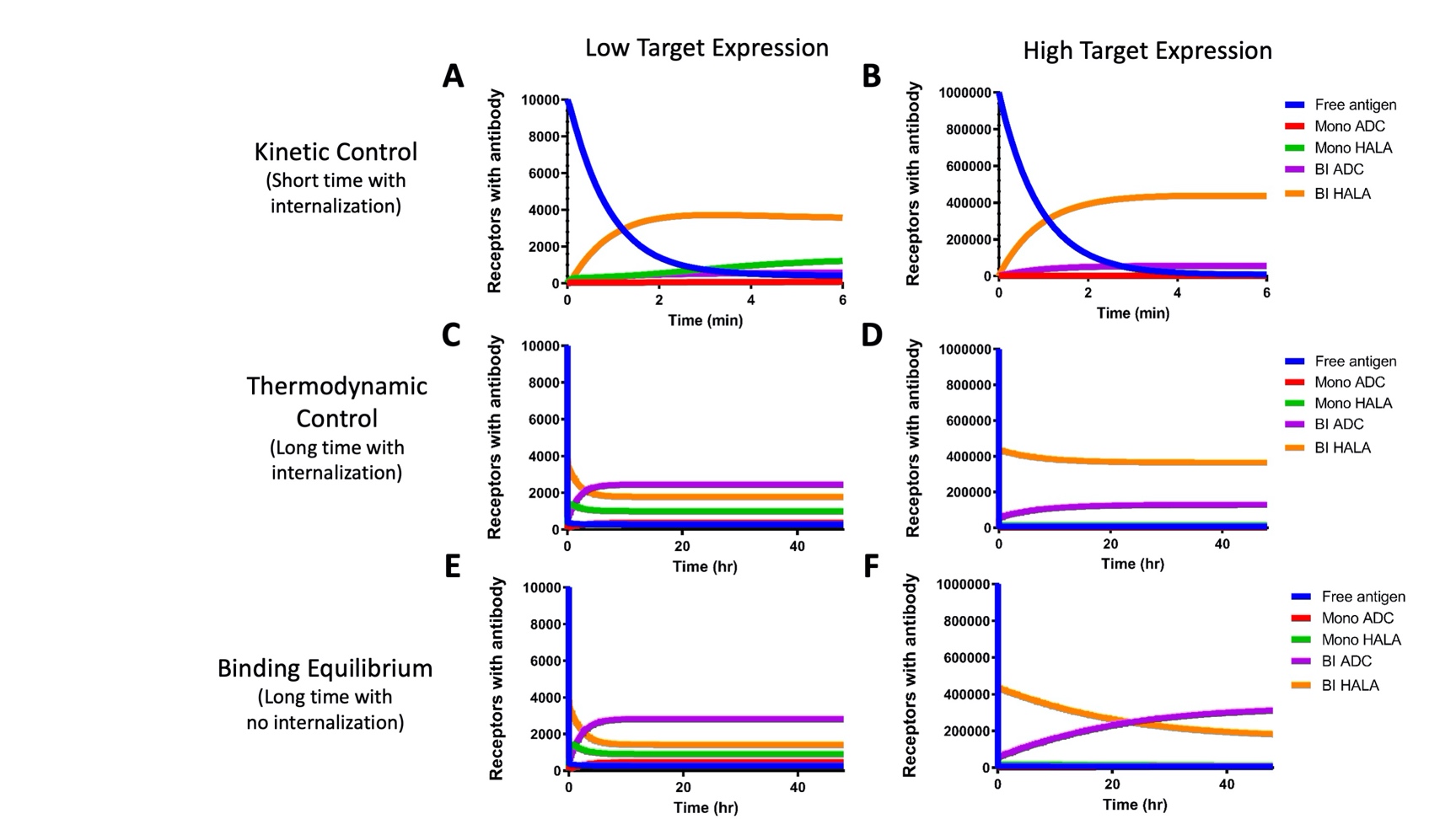


**Figure S2: Thermodynamic and Kinetic Control in Simulations.** During short times (kinetic control) HALA antibody binding outcompetes the ADC because of the higher HALA antibody concentration and similar on-rates for ADC and antibody. Equilibrium is not reached within the 6 minute simulation time for either low expressing cells (A) or high expressing cells (B). At long times (48 hr simulation time), the ADC begins to compete with the HALA antibody; however, competition is dependent on the expression level. For low expressing systems, the ADC rapidly outcompetes the HALA antibody resulting in the majority of antigen bound by ADC (C). For high expressing systems, the avidity of the HALA antibody results in a slow approach to thermodynamic equilibrium. In fact, internalization removes the bound complex from the cell surface before the ADC can outcompete the HALA antibody, resulting in permanent high HALA antibody binding (D). If there were no internalization, both the low (E) and high (F) expression systems approach thermodynamic equilibrium binding with the ADC dominating the surface binding.


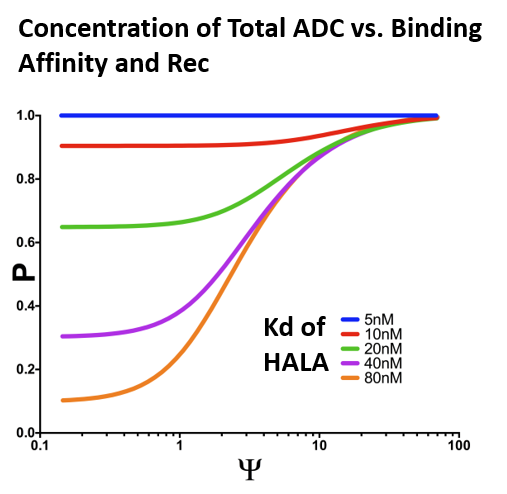


**Figure S3: P vs Ѱ graphs at varying HALA antibody K_d_.** While higher of values of Ѱ converge to a P value of 1, individual parameters such as K_d_ can affect P values at lower Ѱ. For example, the changing HALA dose is plotted above at varying affinities (K_d_). At low affinities (e.g. 80 nM), the HALA antibody is unable to block uptake faster than the internalization rate, so the ADC outcompetes almost all the HALA antibody. However, as the affinity of the HALA antibody increases (decreasing K_d_), the HALA antibody more effectively blocks the ADC at equilibrium. When the HALA affinity equals that of the ADC (5 nM), P always equals 1 since the high ADC affinity assumption fails.


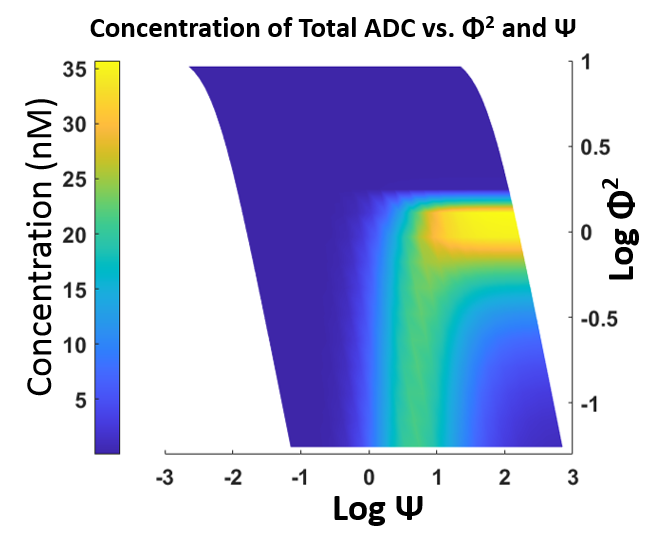


**Figure S4. Total Concentration of ADC at center of spheroid vs Ѱ and Φ^2^.** If the HALA antibody concentration is too high (small Φ^2^), Ѱ is between 1 and 10 (log 0 to log 1) to balance competition. When the optimal amount of HALA is given (to achieve Φ^2^ ~1), Ѱ is > 10 for efficient tissue penetration.


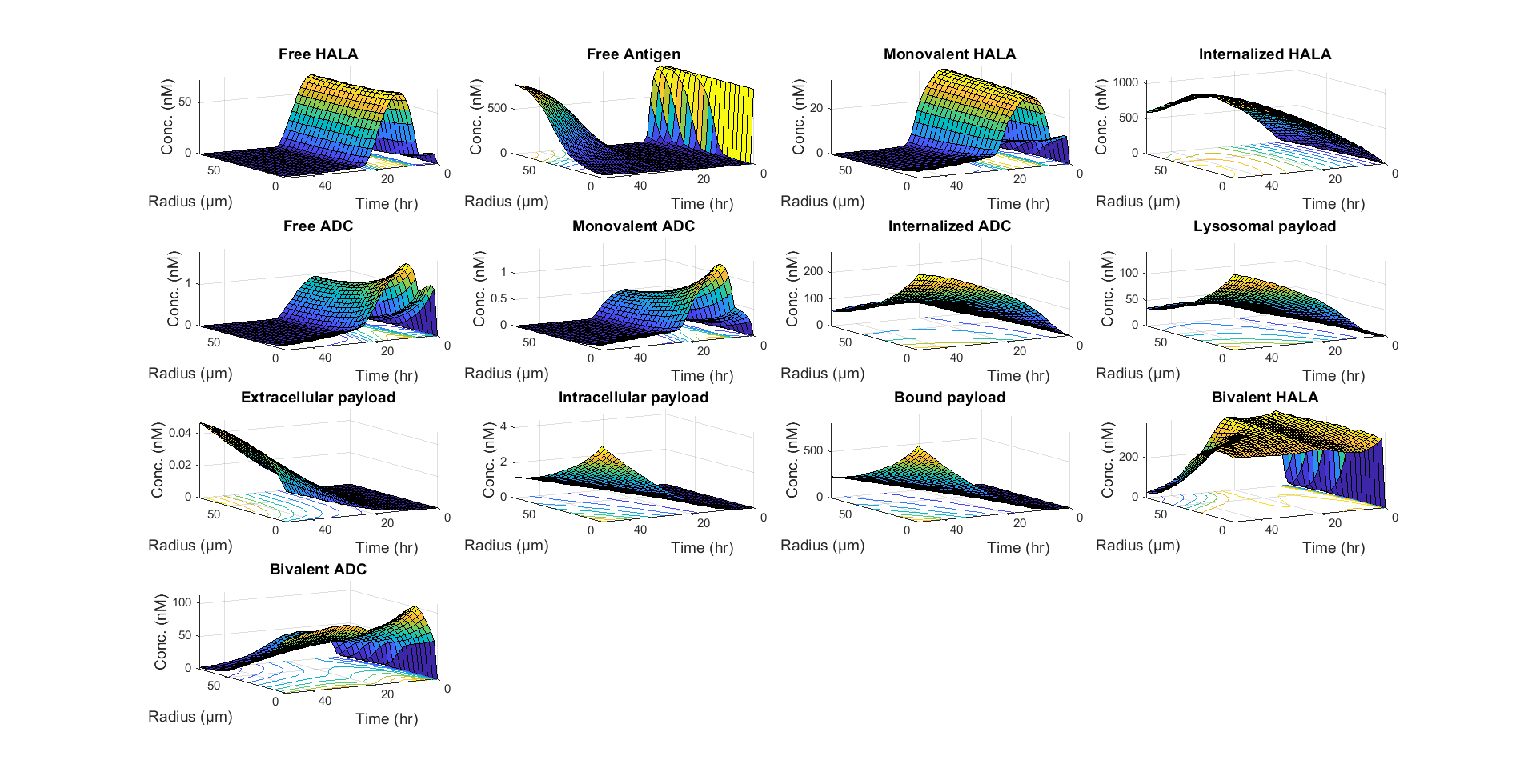


**Figure S5: Krogh Cylinder simulations of a non-bystander payload at 8:1 dose of HALA and ADC with a 3.6mg/kg ADC dose.** Bound Payload is a function of amount of bound ADC and the amount of internalized Payload. Although Bound ADC declines over time, the amount of internalized ADC increases over time, thus the Bound Payload increases.


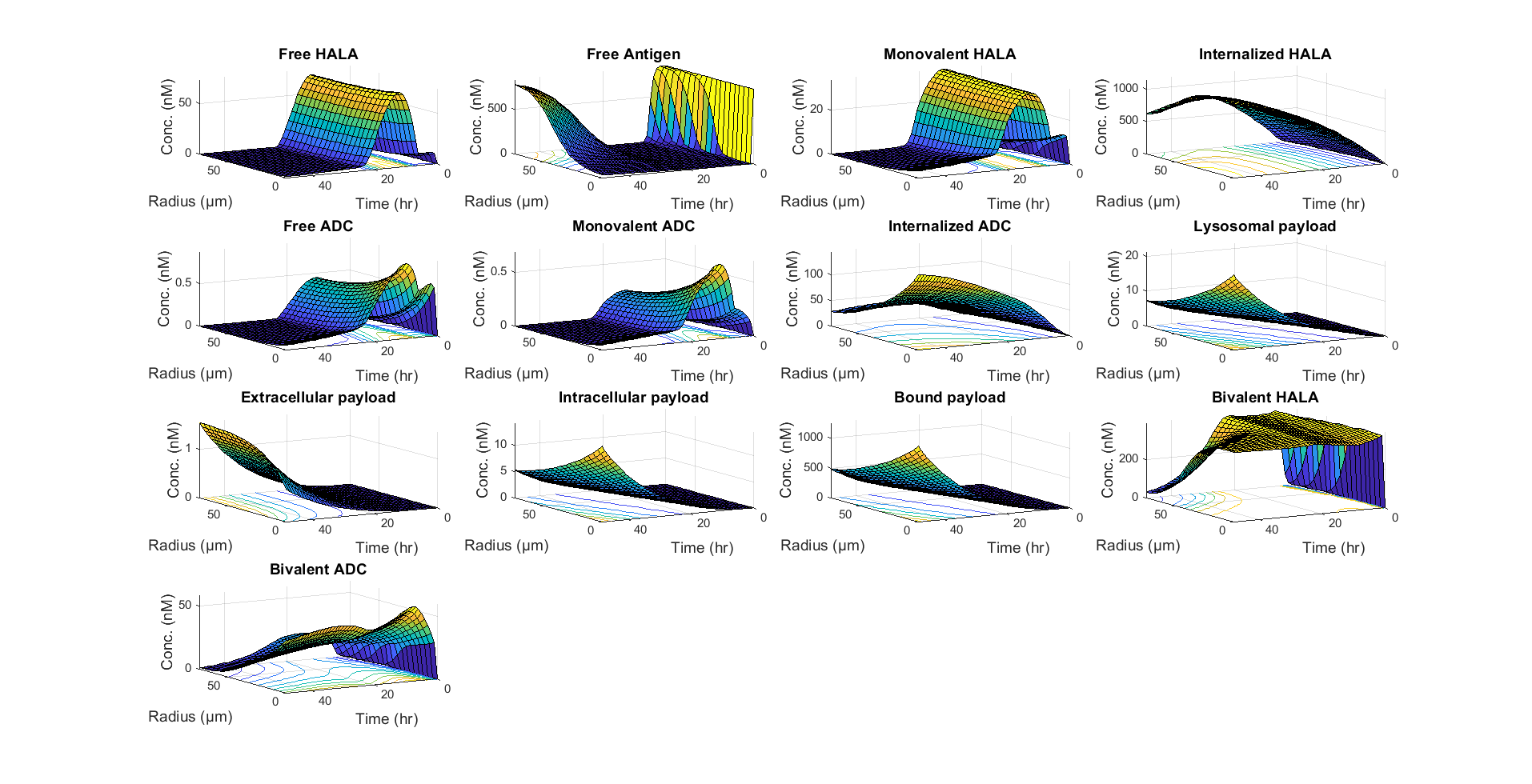


**Figure S6: Krogh Cylinder simulations of a bystander payload at 16:1 dose of HALA and ADC with a 1.8mg/kg T-MMAE dose.** The HALA dose was increased to maintain a total dose that overcomes internalization. Bound Payload is a function of amount of bound ADC and the amount of internalized Payload. Although Bound ADC declines over time, the amount of internalized ADC increases over time, thus the Bound Payload increases. However, due to the bystander effects, the Bound payload amount increases.

**
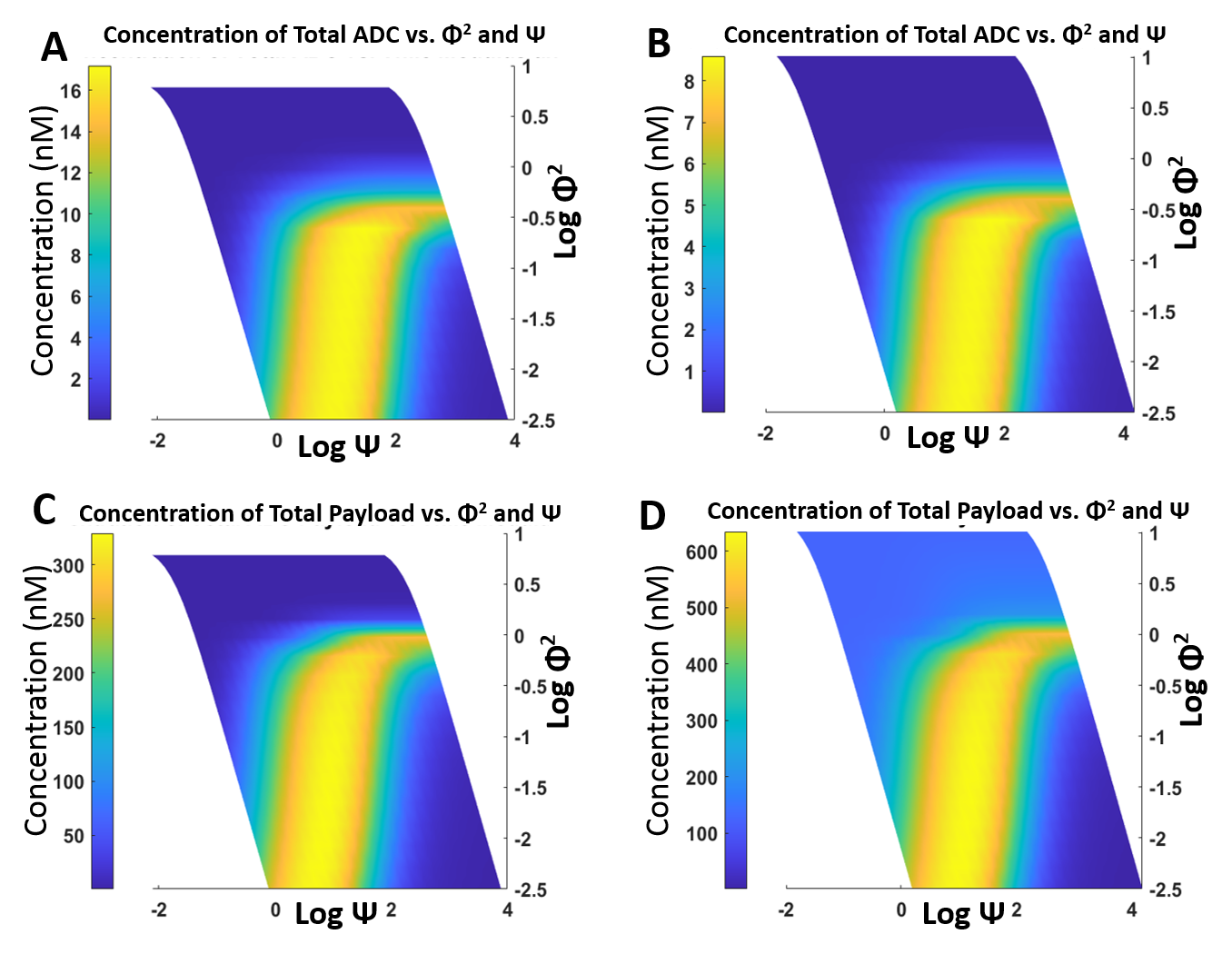
**

**Figure S7. Distribution of ADC and Payload of co-administered ADC with HALA antibody showing changing concentration and binding affinity as a function of Ѱ and Φ^2^ in Krogh Cylinder simulations after 48 hours** (A) Distribution of ADC at dose of 3.6mg/kg with DM1 payload (non-bystander) (B) Distribution of ADC at dose of 1.8mg/kg with bystander (MMAE) payload. (C) Payload of ADC with non-bystander payload. (D) Payload of ADC with bystander payload. The light blue region above the yellow has a higher concentration due to MMAE bystander payload distribution. Due to differences in internalization between the ADC and Payload ADC has a smaller Φ^2^ at larger Ѱ values, respective to the payload. However, when Φ^2^ is close to 1. Ѱ is > 1 for good penetration.

**
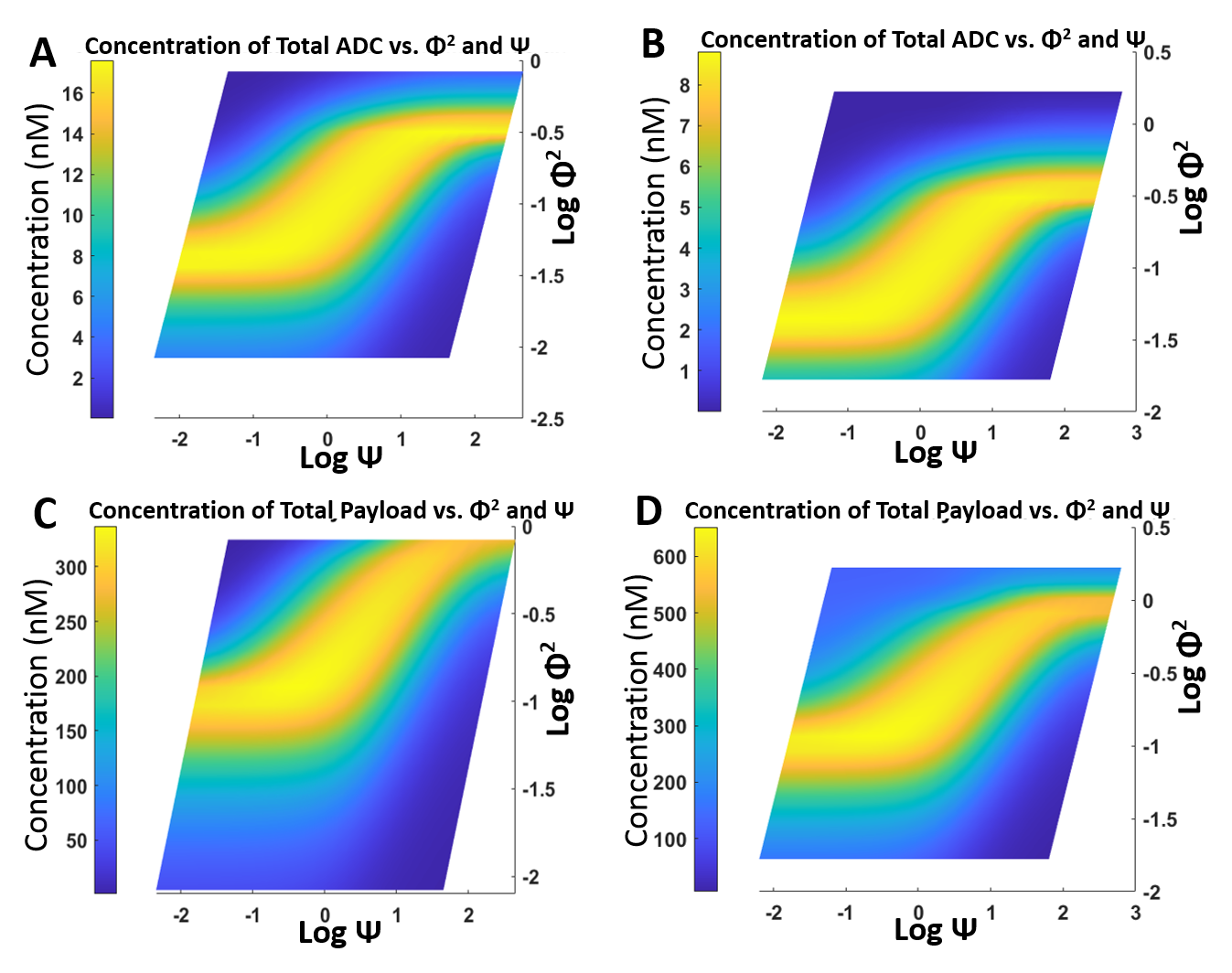
**

**Figure S8. Design of Optimal HALA with changing Antibody Dose and Affinity as a function of Ѱ and Φ^2^ in Krogh Cylinder simulations after 48 hours** (A) Distribution of ADC at dose of 3.6mg/kg with DM1 payload (non-bystander) (B) Distribution of ADC at dose of 1.8mg/kg with bystander (MMAE) payload. (C) Payload of ADC with non-bystander payload. (D) Payload of ADC with bystander payload. The light blue region above the yellow has a higher concentration due to MMAE bystander payload distribution. Due to differences in internalization between the ADC and Payload ADC has a smaller Φ^2^ at larger Ѱ values, respective to the payload. However, when Φ^2^ is close to 1. Ѱ is > 1 for good penetration.

**References:**

1. Bostrom, J., Haber, L., Koenig, P., Kelley, R. F. & Fuh, G. High affinity antigen recognition of the dual specific variants of herceptin is entropy-driven in spite of structural plasticity. *PLoS One* **6**, e17887, doi:10.1371/journal.pone.0017887 (2011).
2. Landry, J. P., Ke, Y., Yu, G. L. & Zhu, X. D. Measuring affinity constants of 1450 monoclonal antibodies to peptide targets with a microarray-based label-free assay platform. *J Immunol Methods* **417**, 86-96, doi:10.1016/j.jim.2014.12.011 (2015).
3. Thurber, G. M. & Weissleder, R. A systems approach for tumor pharmacokinetics. *PLoS One* **6**, e24696, doi:10.1371/journal.pone.0024696 (2011).
4. Wittrup, K. D., Tidor, B., Hackel, B. J. & Sarkar, C. A. *Quantitative fundamentals of molecular and cellular bioengineering*. (MIT Press, 2020).
5. Tang, Y. *et al.* Regulation of antibody-dependent cellular cytotoxicity by IgG intrinsic and apparent affinity for target antigen. *J Immunol* **179**, 2815-2823, doi:10.4049/jimmunol.179.5.2815 (2007).
6. Khera, E., Cilliers, C., Bhatnagar, S. & Thurber, G. M. Computational transport analysis of antibody-drug conjugate bystander effects and payload tumoral distribution: implications for therapy. *Molecular Systems Design & Engineering* **3**, 73-88 (2018).
7. Thurber, G. M., Zajic, S. C. & Wittrup, K. D. Theoretic criteria for antibody penetration into solid tumors and micrometastases. *J Nucl Med* **48**, 995-999, doi:10.2967/jnumed.106.037069 (2007).
8. Hendriks, B. S., Opresko, L. K., Wiley, H. S. & Lauffenburger, D. Coregulation of epidermal growth factor receptor/human epidermal growth factor receptor 2 (HER2) levels and locations: quantitative analysis of HER2 overexpression effects. *Cancer Res* **63**, 1130-1137 (2003).
9. Austin, C. D. *et al.* Endocytosis and sorting of ErbB2 and the site of action of cancer therapeutics trastuzumab and geldanamycin. *Mol Biol Cell* **15**, 5268-5282, doi:10.1091/mbc.e04-07-0591 (2004).
10. Thurber, G. M. & Wittrup, K. D. Quantitative spatiotemporal analysis of antibody fragment diffusion and endocytic consumption in tumor spheroids. *Cancer Res* **68**, 3334-3341, doi:10.1158/0008-5472.CAN-07-3018 (2008).
11. Khera, E. *et al.* Quantifying ADC bystander payload penetration with cellular resolution using pharmacodynamic mapping. *Neoplasia* **23**, 210-221, doi:10.1016/j.neo.2020.12.001 (2021).
12. Baker, J. H. *et al.* Direct visualization of heterogeneous extravascular distribution of trastuzumab in human epidermal growth factor receptor type 2 overexpressing xenografts. *Clin Cancer Res* **14**, 2171-2179, doi:10.1158/1078-0432.CCR-07-4465 (2008).
13. Hilmas, D. E. & Gillette, E. L. Morphometric analyses of the microvasculature of tumors during growth and after x-irradiation. *Cancer* **33**, 103-110, doi:10.1002/1097-0142(197401)33:1<103::aid-cncr2820330116>3.0.co;2-7 (1974).
14. Thurber, G. M. & Weissleder, R. A systems approach for tumor pharmacokinetics. *PLoS One* **6**, e24696, doi:10.1371/journal.pone.0024696 (2011).
15. Yuan, F. *et al.* Vascular permeability in a human tumor xenograft: molecular size dependence and cutoff size. *Cancer Res* **55**, 3752-3756 (1995).
16. Ferl, G. Z., Kenanova, V., Wu, A. M. & DiStefano, J. J. A two-tiered physiologically based model for dually labeled single-chain Fv-Fc antibody fragments. *Mol Cancer Ther* **5**, 1550-1558, doi:10.1158/1535-7163.MCT-06-0072 (2006).
17. Green, E. L. Biology of the laboratory mouse. (1966).
18. Maass, K. F., Kulkarni, C., Betts, A. M. & Wittrup, K. D. Determination of Cellular Processing Rates for a Trastuzumab-Maytansinoid Antibody-Drug Conjugate (ADC) Highlights Key Parameters for ADC Design. *AAPS J* **18**, 635-646, doi:10.1208/s12248-016-9892-3 (2016).
19. Pruijn, F. B., Patel, K., Hay, M. P., Wilson, W. R. & Hicks, K. O. Prediction of tumour tissue diffusion coefficients of hypoxia-activated prodrugs from physicochemical parameters. *Australian journal of Chemistry* **61**, 687-693 (2008).
20. Zhang, L., Bhatnagar, S., Deschenes, E. & Thurber, G. M. Mechanistic and quantitative insight into cell surface targeted molecular imaging agent design. *Sci Rep* **6**, 25424, doi:10.1038/srep25424 (2016).
21. Singh, A. P. *et al.* Evolution of Antibody-Drug Conjugate Tumor Disposition Model to Predict Preclinical Tumor Pharmacokinetics of Trastuzumab-Emtansine (T-DM1). *AAPS J* **18**, 861-875, doi:10.1208/s12248-016-9904-3 (2016).
22. Bhatnagar, S., Deschenes, E., Liao, J., Cilliers, C. & Thurber, G. M. Multichannel imaging to quantify four classes of pharmacokinetic distribution in tumors. *J Pharm Sci* **103**, 3276-3286, doi:10.1002/jps.24086 (2014).

Krol, A., Maresca, J., Dewhirst, M. W. & Yuan, F. Available volume fraction of macromolecules in the extravascular space of a fibrosarcoma: implications for drug delivery. *Cancer Res* **59**, 4136-4141 (1999).
